# *cfNOMe* — A single assay for comprehensive epigenetic analyses of cell-free DNA

**DOI:** 10.1186/s13073-020-00750-5

**Published:** 2020-06-24

**Authors:** Florian Erger, Deborah Nörling, Domenica Borchert, Esther Leenen, Sandra Habbig, Michael S. Wiesener, Malte P. Bartram, Andrea Wenzel, Christian Becker, Mohammad R. Toliat, Peter Nürnberg, Bodo B. Beck, Janine Altmüller

**Affiliations:** 1 Cologne Center for Genomics, University of Cologne, Cologne, Germany; 2 Institute of Human Genetics, Faculty of Medicine and University Hospital Cologne, Cologne, Germany; 3 Center for Molecular Medicine Cologne (CMMC), University of Cologne, Cologne, Germany; 4 Department of Nephrology, Transplantation and Medical Intensive Care, University Witten/Herdecke, Medical Center Cologne-Merheim, Cologne, Germany; 5 Department of Pediatrics, Faculty of Medicine and University Hospital Cologne, Cologne, Germany; 6 Department of Nephrology and Hypertension, Friedrich-Alexander University Erlangen-Nürnberg, Erlangen, Germany; 7 Department II of Internal Medicine, Faculty of Medicine and University Hospital Cologne, Cologne, Germany

**Keywords:** cfNOMe, Cell-free DNA, Methylome, Nucleosome footprints, Analysis software

## Abstract

Cell-free DNA (cfDNA) analysis has become essential in cancer diagnostics and prenatal testing. We present *cfNOMe*, a two-in-one method of measuring cfDNA cytosine methylation and nucleosome occupancy in a single assay using non-disruptive enzymatic cytosine conversion and a custom bioinformatic pipeline. We show that enzymatic cytosine conversion better preserves cfDNA fragmentation information than does bisulfite conversion. Whereas previously separate experiments were required to study either epigenetic marking, *cfNOMe* delivers reliable results for both, enabling more comprehensive and inexpensive epigenetic cfDNA profiling. *cfNOMe* has the potential to advance biomarker discovery and diagnostic usage in diseases with systemic perturbations of cfDNA composition.

## Background

Cell-free DNA (cfDNA) maintains the epigenetic signatures of its tissues-of-origin [1–3]. Studies of genome-wide tissue-specific methylation signatures from cfDNA have so far shown promise in the deconvolution (i.e., the identification and quantification of contributing fractions) of its tissues-of-origin. Bisulfite cytosine conversion (BCC)-based methylation array studies [4], as well as next-generation sequencing (NGS) [2, 5, 6] and targeted pyrosequencing [7] studies, have demonstrated that the composition of cfDNA, and hence its epigenetic signature, varies in states of health and disease. cfDNA is primarily released by dying cells. A minimally invasive method to identify and quantify its tissues-of-origin could be used to take a “snapshot” of ongoing tissue damage throughout the organism at the moment of sample collection. The translational applications of such an approach cannot be overrated if sufficient resolution can be achieved.

Current diagnostic applications of cfDNA, like “liquid biopsy” [8], non-invasive prenatal testing [9], or detection of transplant rejection [10], mainly leverage genotypic differences and/or copy number variation. Epigenetic studies of cfDNA, on the other hand, are expected to be informative in the large majority of patients, as shown in methylation signature-based studies in patients with myocardial infarction [11] or relapsing-remitting multiple sclerosis [12]. Urine-derived cfDNA can also be studied directly to detect infections of the urinary tract [13].

A second layer of indirectly measurable epigenetic information in cfDNA is nucleosome occupancy (NO). Nucleosomes in the cfDNA’s tissue-of-origin locally protect against endonuclease cleavage during apoptosis, thereby leading to a biased cfDNA fragmentation pattern, from which NO is inferred [1]. This fragmentation pattern is apparent in aligned NGS data, but not detectable in microarray- or pyrosequencing-based approaches. Such measurements of NO have been successfully used to infer the gene expression [14] and regulation [3] landscapes of the cfDNA’s tissue-of-origin. Most recently, a similar approach was reported to identify cell lineage-specific transcription factor binding for the prediction of tumor subtypes in prostate cancer [15].

The combined NGS analysis of Nucleosome Occupancy and Methylation profiles (NOMe-Seq [16]) has been possible in nuclear (i.e., non-cell-free) DNA for some years. This method targets open chromatin, however, and is therefore not applicable to cfDNA. Other methods of jointly profiling independent epigenetic attributes (methylation and chromatin conformation) in nuclear DNA have also recently been described [17].

Bisulfite cytosine conversion has been the gold standard in DNA methylation analysis for decades [18], but causes significant degradation [19–21], fragmentation [22], and GC biases [23, 24]. New, alternative methods of chemical cytosine conversion seek to address these issues [25]. In addition, enzymatic cytosine conversion (ECC) was recently reported using APOBEC3A (ACE-Seq [26]) that enables the precise quantification of 5-hydroxymethylcytosine (5hmC), but not 5-methylcytosine (5mC). We therefore selected a novel method of enzymatic cytosine conversion (NEBNext® EM-Seq® [27], New England Biolabs, Ipswich, MA, USA) that is capable of detecting both 5mC and 5hmC (Additional file 1: Fig. S1) and allows whole-genome methylation studies with small amounts of input DNA and a decreased coverage bias against GC-rich regions (Additional file 1: Fig. S2). In this study, we (1) compare the performance of ECC and BCC in the context of cfDNA methylation and nucleosome occupancy analyses and (2) perform ECC on the cfDNA of healthy controls and patients with acute kidney injury (AKI), acute kidney graft dysfunction (AGD), or chronic kidney disease to identify perturbations of cellular turnover in the affected tissues. Using a custom bioinformatic pipeline, we are able to simultaneously measure both nucleosome occupancy and methylation profiles from cfDNA. We term our assay “cell-free DNA-based Nucleosome Occupancy and Methylation profiling” (*cfNOMe*).

## Methods

### Patient recruitment

Patients admitted to collaborating clinical departments with kidney disease were contacted and—upon securing written informed consent for study participation—between one and three fresh urine and venous blood samples were collected during routine diagnostic blood drawings to be further processed as described below. The study was approved by the Ethics Committee of the Medical Faculty of the University of Cologne, Germany (study registration ID 15-215).

### Cell-free DNA isolation from urine and plasma

Urine samples were freshly collected from patients and healthy controls in falcon tubes and directly—within 30 min—processed by centrifugation at 1600*g* for 10 min at RT to separate cell debris from supernatant. The supernatant was centrifuged a second time for 20 min at 3200*g* at RT to remove the remaining cellular components.

Blood samples were freshly collected in cell-free DNA BCT CE collection tubes (STRECK, La Vista, NE, USA) containing K_3_ EDTA. It was then directly processed by centrifugation at 1500*g* for 12 min at 4 °C to separate red blood cells from plasma. The supernatant was centrifuged a second time for 12 min at 1500*g*, RT to remove remaining cellular components. We aimed for a minimal delay between blood drawing and centrifugation of ≤ 2 h, which was achieved for the majority of samples. Samples P30 and P31 had the highest processing delay of ~ 48 h. The manufacturer of DNA BCT CE collection tubes guarantees cfDNA and gDNA stability between 6 and 37 °C for up to 14 days. Urine supernatant and plasma were stored at − 80 °C (at least overnight) before being further processed.

Blood and urine samples from healthy control K17 were collected at 8:00 am, noon, and 9:30 pm on subsequent days. This was done to exploratively study the temporal variability of cfDNA composition in healthy individuals.

For cfDNA isolation, urine and plasma samples were thawed on ice for 1 h and processed according to the QIAamp Circulating Nucleic Acid Kit Handbook (Qiagen, Hilden, Germany). The protocol was modified as follows: The input volume of urine was increased from 4 to 10 ml, and buffer volumes were adjusted accordingly. Also, the incubation time for elution with AVE buffer was increased from 3 to 30 min.

After sample preparation, cfDNA was quantified via a Qubit 3.0 fluorometer (Thermo Fisher Scientific, Waltham, MA, USA) using the high sensitivity assay kit. Fragment size determination was performed using the 4200 TapeStation (Agilent, Santa Clara, CA, USA) instrument according to manufacturer standards.

### Converted library preparation

**Fig. 1:**
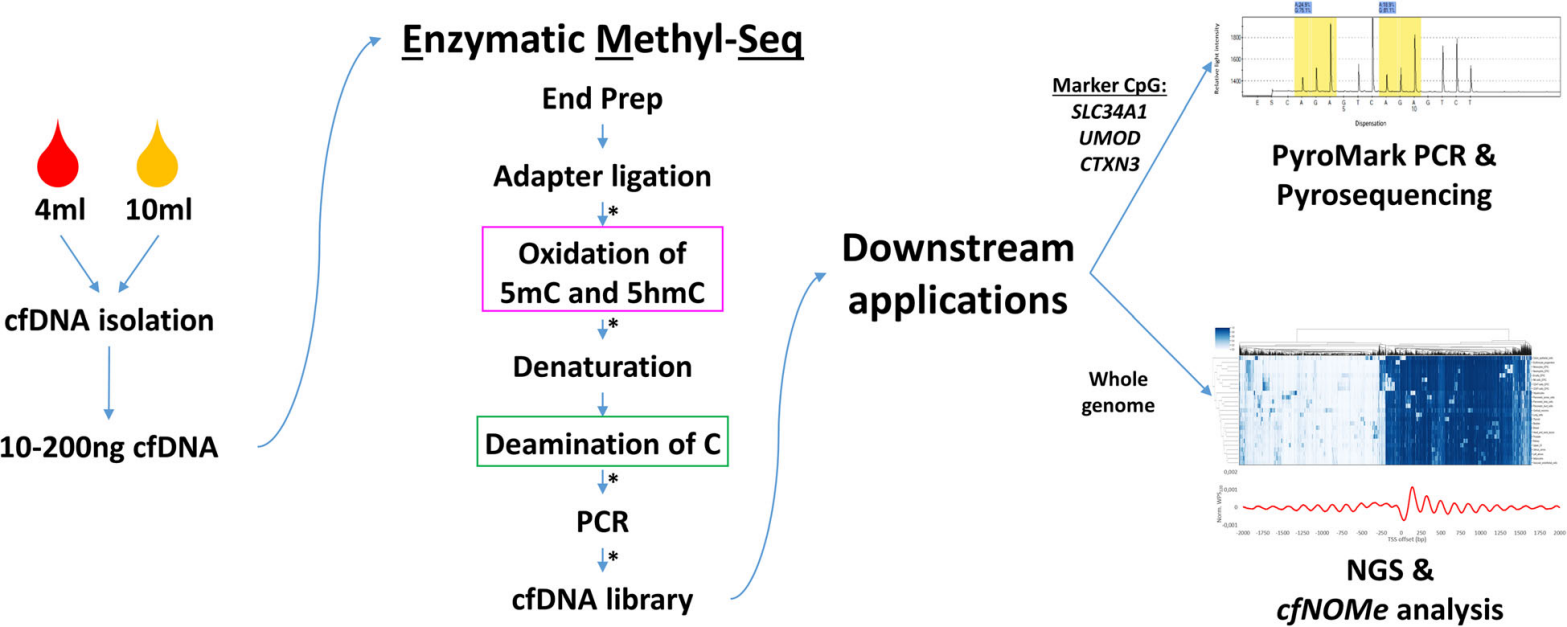
Workflow for enzymatic cytosine conversion from cfDNA isolation to pyrosequencing. Shown is the workflow, starting with cfDNA isolation from urine supernatant or plasma via a vacuum pump and spin column-based method. After a subsequent library preparation step, conversion of cytosines is performed enzymatically, followed by the desired downstream analyses. Purple and green boxes highlight crucial steps in enzymatic conversion. The asterisk indicates clean-up steps

Library preparation was performed according to the NEB EM-Seq protocol (New England Biolabs, Ipswich, MA, USA) with the following modifications: No fragmentation was done for cfDNA samples. After library preparation, cfDNA was quantified via a Qubit 3.0 fluorometer (Fig. 1; Additional file 1: Tables S1-S3).

In the replication cohort, fully methylated pUC19 control DNA and unmethylated lambda DNA were fragmented to 180 nt by sonication using a bioruptor (Diagenode, Seraing, Belgium). 0.01 ng of pUC19 control DNA and 0.2 ng lambda DNA per 10 ng sample DNA was added to each sample. All samples were library prepped using the above protocol. However, the healthy control samples K19, K20, and K21 were split up after the adapter ligation. One replicate of each was then converted using the NEB EM-Seq kit, the other with the EpiTect Bisulfite Kit (QIAGEN, Hilden, Germany) according to the standard protocol. For direct comparison, the respective replicates received exactly the same protocol and amplification; the type of conversion was the only difference (Additional file 1: Tables S1-S3).

### PyroMark PCR

To amplify regions of interest in cfDNA and further determine its methylation status, PyroMark PCR was performed (Additional file 1: Tables S4-S6). Six nanograms of cfDNA was used for each PCR reaction. *SLC34A1*, *UMOD*, and *CTXN3* were studied. The genomic positions analyzed in *SLC34A1* were (hg19) chr5:176812116 (locus 1), chr5:176812123 (locus 2), and chr5:176812139 (locus 3); in *UMOD* chr16:20364262 (locus 1) and chr16:20364284 (locus 2); and in *CTXN3* chr5:126988758 (locus 1). Loci of interest were identified within these genes in a manual screen of differentially methylated regions near genes with highly enriched kidney expression. In short, we inspected publicly available tissue-specific whole-genome bisulfite sequencing (WGBS) data tracks in the genomic vicinity of genes with a known kidney-specific expression pattern as listed in the “Tissue enriched” and “Group enriched” categories on the Human Protein Atlas resource [28]. Non-cultured, non-fetal tissue hg19 WGBS tracks stored in the International Human Epigenome Consortium Data Portal [29] were compared. The abovementioned loci of interest showed kidney-specific hypomethylation and were thus selected for our initial enzymatic conversion and pyrosequencing experiments. As expected, hypermethylation was observed in blood-derived cfDNA for all loci (between average values of 81.6% ± 3.4% for *CTXN3* and 98.5% ± 1.9% for *SLC34A1* locus 3), while the methylation levels in urine-derived cfDNA were notably lower (between 49.8% ± 6.5% for *CTXN3* and 68.7% ± 5.9% for *SLC34A1* locus 3). These data were then used to quantify the correlation between pyrosequencing methylation measurements and NGS methylation measurements.

### Pyrosequencing

0.36 μl of sequencing primer (10 μM) and 11.64 μl AB buffer (1x) per well were added to a pyrosequencing plate.

Isolation of the single-stranded pyrosequencing template was performed by mixing each PCR probe with 70 μl sepharose beads in binding buffer for 5 min at 1000 rpm to allow streptavidin beads to bind the biotin-labeled strand.

The vacuum prep station was set up with H_2_O, 70% ethanol, denaturation buffer, and 1x wash buffer (200 ml each). Mixed cfDNA-bead-samples were then soaked up by the probes and washed with EtOH70% to dispose residual salts and unlabeled cfDNA, then with denaturation buffer to denature amplicons and remove the unlabeled cfDNA strands from the sample, leaving the ssDNA pyrosequencing template. Next, the probes were washed with 1x wash buffer to allow the pyrosequencing reaction to proceed at proper conditions. Finally, vacuum was switched off and probes were lowered into the priming plate containing the annealing primer-buffer-mix and agitated vigorously to facilitate beads dropping from the filters into the buffer-primer-mix. The pyrosequencing plate was then incubated at 85 °C for 2 min to eliminate any secondary structures in the single-stranded template that may interfere with primer annealing or enzymatic addition of nucleotides. Samples were then analyzed via the PSQ HS 96 device and software (QIAGEN, Hilden, Germany).

### Whole-genome sequencing library preparation of donor gDNA

The libraries were prepared and size selected by using the Illumina® TruSeq PCRfree® (Illumina, San Diego, CA, USA) DNA Sample Preparation Kit and Agencourt AMPure XP beads (Beckman Coulter, Indianapolis, IN, USA), starting with 1.2 μg input DNA and followed by one cycle of PCR to complete adapter structure.

### Sequencing of WGS and converted libraries

The cfDNA library quality parameters were validated with the Agilent 2200 TapeStation, and library quantification was done with qPCR. Using Illumina NovaSeq 6000 devices, we generated between 64.6 and 96.8 Gb (per WGS library), between 192.8 and 489.7 Gb (per *cfNOMe* library in the discovery cohort), and between 105.3 and 262.3 Gb (per *cfNOMe*/bisulfite-converted library in the replication cohort) paired-end 150-bp data [30]. These yields corresponded to an expected coverage of ~ 20–30-fold, ~ 50–130-fold, and ~ 30–40-fold respectively (Additional file 1: Tables S7 and S8).

### Bioinformatic processing

After standard demultiplexing and adapter trimming of raw NGS data, the FASTQ files for each replicate were aligned against the hg19 human reference genome using the Bismark (v0.20.0) methylation-sensitive alignment tool [31] in conjunction with bowtie2 (v2.3.4.1). The mapping efficiency metrics (Additional file 1: Fig. S3) were taken from the Bismark alignment report. In the replication cohort, the larger bisulfite-converted datasets were downsampled to the size of the corresponding EMseq dataset prior to alignment.

The aligned BAM files were sorted and indexed using samtools (v1.7, [32]). An additional merged BAM for each sample was created from its respective replicate subfiles.

### Downsampling

Each downsampled dataset was generated from the complete, merged alignment file using the BBmap reformat tool’s (v38.75) samplereadstarget parameter.

### Calculation of renal cfDNA fraction from donor SNPs

We first performed whole-genome sequencing (WGS) of kidney donor DNA and, using the samtools *mpileup* function and bcftools (v1.7), identified positions where the respective donor was either homozygous (donor for patient P27) or heterozygous (donor for patient P29) for a single nucleotide A>T or T>A transversion. We then similarly evaluated each position in the existing blood-derived *cfNOMe* datasets of the transplant recipients. We excluded positions at which a variant call of heterozygous or homozygous was made in the recipient under the assumption that the transplant cfDNA fraction in blood would be low enough to not cause erroneous heterozygous calls in the recipient cfDNA dataset. The so chosen positions (*n* = 1326 for P27 and *n* = 776 for P29) were then extracted from the respective blood- and urine-derived *cfNOMe* datasets and the percentage variant alleles at each position calculated. These allele fractions were averaged to calculate the most probable graft-derived, i.e., renal cfDNA fraction in patient P27’s blood and urine. Because of patient P29’s first-degree relatedness to their donor, only positions at which the donor was heterozygous for a variant allele could be compared. The allele fractions were therefore doubled to calculate the renal cfDNA fraction in blood- and urine-derived cfDNA (Additional file 1: Fig. S4).

### Nucleosome footprinting analyses

The transcription start site (TSS) coordinates for all RefSeq-listed genes were accessed using the UCSC Table Browser. For each TSS, the windowed protection score with the chosen window size (e.g., 120 bp: WPS_120_) was calculated for every nucleotide within 5 kb up- and downstream using the previously generated alignment files and batch processing of the input coordinates. The resulting WPS was normalized to the number of fragments within the analyzed region to control for sequencing depth differences. For aggregate measurements of multiple TSS coordinates, the datasets for each input coordinate were merged and averaged into a single normalized output file with one score for each nucleotide position in the analyzed region (i.e., − 5000 to + 5000 in the case of a 10-kb region). An additional normalization step was performed by calculating a running average within ± 500 bp of each nucleotide position and subtracting this from the respective WPS, thereby normalizing the data to a running 1-kb mean of zero. From this, a secondary output file was generated.

**Fig. 2:**
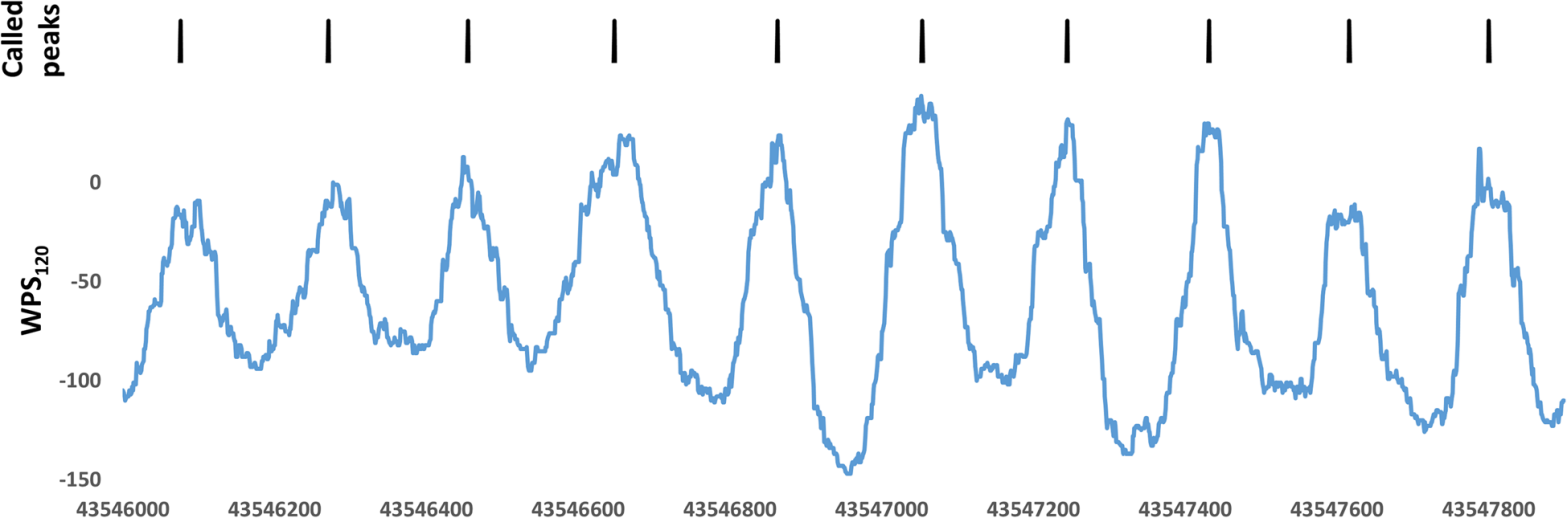
Peak calling accuracy of the described algorithm at a known alpha satellite on chromosome 8. The repetitive genomic structure is mirrored in a well-phased, periodic nucleosome occupancy signal. Coordinates are hg19: 8:43,546,000–43,548,000

WPS peaks were detected for a given genomic region automatically from unmerged, normalized data by identifying continuous sequences of positive scores between 50 and 450 bp in length, uninterrupted by more than 4 negative score values in a row. We then checked which continuous window of scores above the median had the largest total WPS sum and called the peak in the middle of this window (Fig. 2). Interpeak distances (IPDs) were subsequently extracted and tabulated for distances up to 1 kb.

For the comparison of IPDs between high- and low-expression genes, we used a Wilcoxon rank-sum test (Mann-Whitney *U* test) to check for differences in IPD distribution.

**Fig. 3:**
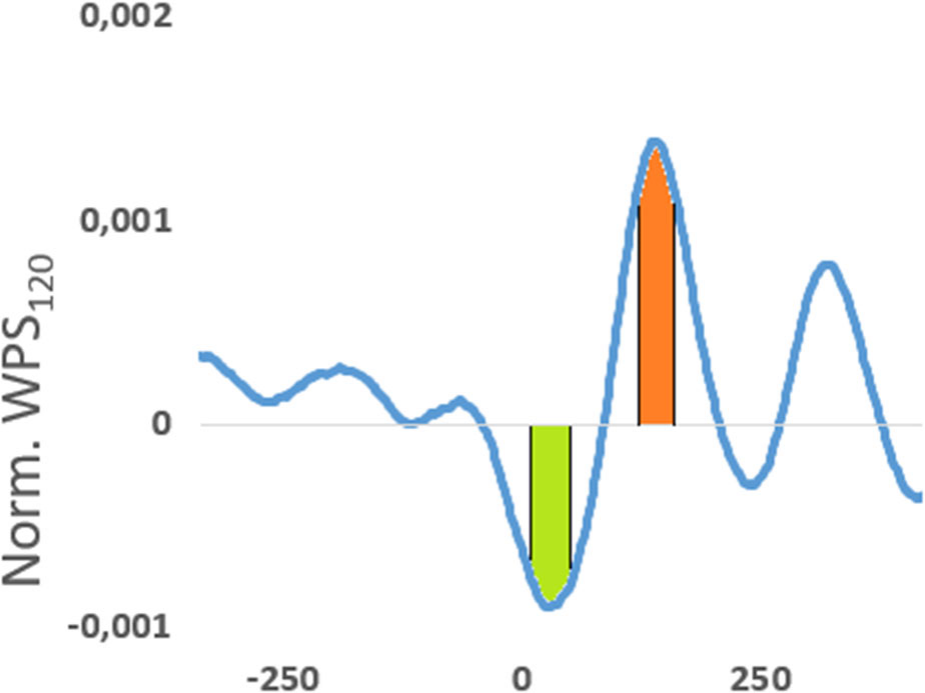
Quantification of nucleosome organization strength. The summed WPS_120_ for all points in the green shaded area (∑*P*_10..60_) is subtracted from the summed WPS_120_ for all points in the red shaded area (∑*P*_135..185_). Different TSS-offsets must be used if a different WPS window length is chosen. A high value of this metric represents a more highly organized nucleosome positioning at the studied transcription start sites

For the quantification of nucleosome organization in the immediate vicinity of the transcription start site, the WPS_120_ scores for the most abundant medium length cfDNA fragments (140–200 bp) were normalized and an average value was calculated for bins containing 1000 genes each in descending order of whole blood expression levels. We then scored the amplitude of the nucleosome positioning signal by subtracting the WPS_120_ in the nucleosome-free region from the WPS_120_ at the position of the well-phased + 1 nucleosome (Fig. 3).

### Methylation analysis

For methylation analysis, the Bismark alignment output files were analyzed with the bismark-methylation-extractor tool, ignoring two 5′ nucleotides of read 1 and three 5′ nucleotides as well as one 3′ nucleotide of read 2, for which Bismark reported biased methylation calling patterns (Additional file 1: Fig. S5). Methylation calls over non-CpG cytosines were summarized and calculated genome-wide for all samples and the available fully methylated positive and unmethylated negative controls (Additional file 1: Fig. S6 and S7). Additionally, context-specific methylation results for CpG, CHH, and CHG methylation were extracted for 1000-bp windows throughout the entire genome (see Additional file 1: Fig. S8 for a representative plot of chromosome 2). Bismark output result files were subsequently processed with the bedmap (v2.4.26) tool, and methylation levels for the 7890 informative methylation loci from Moss et al. [4] were thus calculated for each sample.

For the deconvolution of component tissues-of-origin, the sample methylation levels at these 7890 informative loci were compared to the reference values of 25 tissues [4] (Additional file 1: Fig. S9). As long as the number of studied loci is greater than the number of tissues to be deconvoluted, an overdetermined linear system exists. We thus performed linear least squares minimization as implemented in the Python3 scipy.optimize (v1.2.0) library. In short, through iterative optimization, a coefficient was calculated for each tissue so that the sum of the squared deviations between the deconvolution model and the measured cfDNA methylation levels is minimized. An inequality constraint of 1 was imposed so that the sum of all contributing tissues cannot be larger than 100%. Mathematically, we optimize *coeff*_Tissue_ in:

$$ \left[\begin{array}{cccc}{ref}_{1\_\mathrm{Kidney}}& {ref}_{1\_\mathrm{Monocytes}}& \dots & {ref}_{1\_\mathrm{Colon}}\\ {}{ref}_{2\_\mathrm{Kidney}}& {ref}_{2\_\mathrm{Monocytes}}& \dots & {ref}_{2\_\mathrm{Colon}}\\ {}\vdots & \vdots & \ddots & \vdots \\ {}{ref}_{7890\_\mathrm{Kidney}}& {ref}_{7890\_\mathrm{Monocytes}}& \dots & {ref}_{7890\_\mathrm{Colon}}\end{array}\right]\bullet \left[\begin{array}{c}{coeff}_{\mathrm{Kidney}}\\ {}\vdots \\ {}{coeff}_{\mathrm{Colon}}\end{array}\right]=\left[\begin{array}{c} measured\_{meth}_1\\ {} measured\_{meth}_2\\ {}\vdots \\ {} measured\_{meth}_{7890}\end{array}\right] $$

by solving:

$$ \underset{X}{\min }{\left\Vert \left[\begin{array}{cccc}{ref}_{1\_\mathrm{Kidney}}& {ref}_{1\_\mathrm{Monocytes}}& \dots & {ref}_{1\_\mathrm{Colon}}\\ {}{ref}_{2\_\mathrm{Kidney}}& {ref}_{2\_\mathrm{Monocytes}}& \dots & {ref}_{2\_\mathrm{Colon}}\\ {}\vdots & \vdots & \ddots & \vdots \\ {}{ref}_{7890\_\mathrm{Kidney}}& {ref}_{7890\_\mathrm{Monocytes}}& \dots & {ref}_{7890\_\mathrm{Colon}}\end{array}\right]\bullet X-\left[\begin{array}{c} measured\_{meth}_1\\ {} measured\_{meth}_2\\ {}\vdots \\ {} measured\_{meth}_{7890}\end{array}\right]\right\Vert}^2 $$

where *X* represents a vector containing each tissue coefficient and the sum of all coefficients is not greater than 1.

### Methylation level comparisons

Pyrosequencing was done with fully methylated positive control DNA and unmethylated negative control DNA. Pyrosequencing methylation values were then normalized against their respective positive and negative control sample values. For NGS data, conversion events at the previously detailed loci of interest in *SLC34A1*, *UMOD*, and *CTXN3* were counted in the merged sample alignment files and a methylation ratio calculated. Methylation values from pyrosequencing and NGS were then jointly plotted and a linear regression fitted (Additional file 1: Fig. S10). The plot, as well as the regression confidence band, was generated using the Python3 seaborn (v0.9.0) library’s *regplot* function.

### Gene binning

**Fig. 4:**
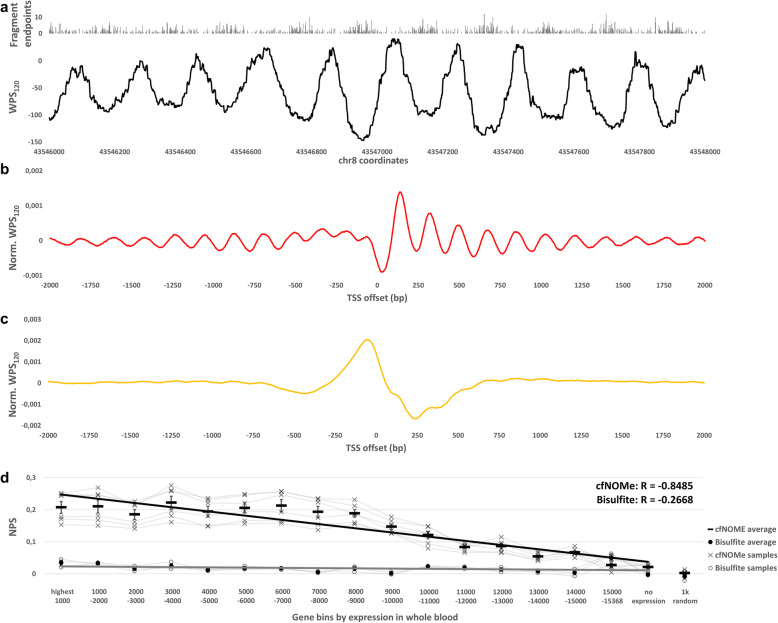
Fragmentation analyses of enzymatically converted cfDNA. Nucleosome occupancy is inferred by calculating, centered on each genomic position, the number of reads with endpoints inside a window of *n* bases and subtracting the number of reads completely spanning this window [1]. The resulting metric is called the windowed protection score (WPS_*n*_). For cfDNA libraries with a fragment length peak at ~ 170 bp, as here, a 120-bp window has been previously used (WPS_120_) [1]. **a** The cfDNA fragmentation shows highly organized nucleosome occupancy in an alpha satellite on chromosome 8 (hg19: chr8:43,546,000–43,548,000). Black line: WPS_120_, gray bars: fragment endpoints. **b** Averaged and normalized WPS_120_ around the TSS of 15,368 genes with positive expression in whole blood. A strong positional pattern within 1 kb of the TSS is visible in blood-derived cfDNA. All fragments are studied. **c** Averaged and normalized WPS_120_ around the TSS of the same 15,368 genes in urine-derived cfDNA. An elongated, “degraded” positional signal within 500 bp of the TSS is visible. All fragments are studied. **d** Quantified nucleosome positioning signal strength around the TSS ranked by gene expression in six healthy controls. Blood-derived cfDNA fragments 140–200 bp in length are studied. The nucleosome positioning signal strength at the TSS is averaged for bins containing 1000 genes in order of descending whole blood expression levels. Shown are 15,368 autosomal genes with non-zero expression in GTEx. The nucleosome positioning signal strength was also calculated for 2447 genes with zero whole blood expression and 1000 random genomic loci, for which it equals close to zero (see the “Methods” section for additional detail). **a**–**c** Merged K16 datasets are analyzed. **d** All merged control datasets are analyzed. Black dashes represent the average of six healthy controls, error bars represent SEM. NPS nucleosome positioning signal

Expression data was accessed from the GTEx portal on 21 March 2019. Data from cell lines were excluded from further analysis. The gene bins for expression in whole blood (Fig. 4) were generated by grouping genes in groups of 1000 by descending order of expression in the “Whole blood” GTEx category. If the expression was identical in whole blood between several genes, the sorting was decided based on global expression as a secondary sorting parameter.

**Fig. 5:**
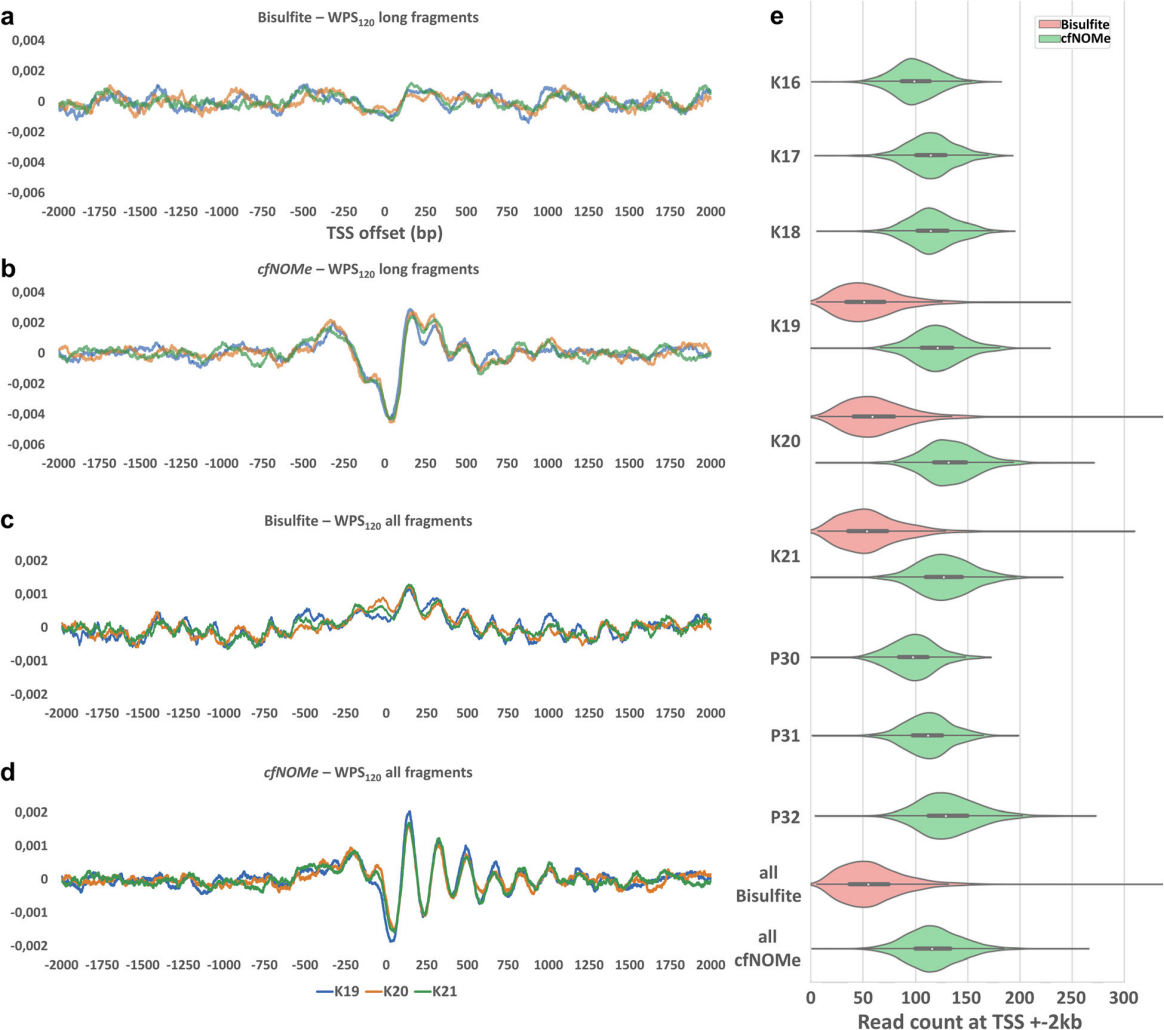
Nucleosome footprinting in bisulfite-treated cfDNA datasets. In a direct comparison between bisulfite-treated and enzymatically converted cfDNA libraries of similar size, the nucleosome positioning is clearly apparent from the enzymatically converted *cfNOMe* dataset, but only barely recognizable in the bisulfite-treated dataset. This difference can be observed when analyzing all fragment lengths in aggregate (**c**, **d**) and is even more pronounced in the long fragments (**a**, **b**). **e** Number of reads aligning to the transcription start site ±2 kb of 8910 autosomal (+)-stranded genes. Dataset sizes were equalized to exactly 100 million aligned reads for this comparison. *cfNOMe* libraries cover these genomic positions twice as well compared to bisulfite-treated libraries. The merged datasets for each specified individual were used for all calculations

Bins for kidney or bladder tissue-enriched genes (Fig. 5) were generated as follows: GTEx genes with low expression in whole blood (< 0.5 transcripts per million) were extracted and grouped together in bins of 1000 by descending expression levels in the tissue-of-interest. The “high expression” groups for the kidney and bladder contained genes with expression levels between 4.5 and 928.0 transcripts per million and 7.8 and 731.3 transcripts per million, respectively.

## Results

### Cohort recruitment

Participants were recruited at two time points. During the first study phase, we generated *cfNOMe* libraries from enzymatically treated blood- and urine-derived cfDNA of six individuals. Three healthy controls and three patients with acute kidney injury or acute kidney graft dysfunction were studied, with a resampling of blood and urine in one patient after improvement of kidney function (*n* = 6 × 2 + 2 = 14 samples). Where possible, sample collection and experiments were performed in triplicate (see Additional file 1: Fig. S11 for details on sample collection and data generation). We thus generated 34 *cfNOMe* replicate libraries (Additional file 1: Table S7), which were processed in parallel and later recombined into 14 merged datasets. During the second recruitment phase, blood samples of three additional healthy control subjects and three patients with chronic kidney disease were collected. Blood-derived cfDNA of the three healthy controls in the second recruitment phase was processed with enzymatic and bisulfite conversion in parallel.

### Enzymatic cytosine conversion reliably measures cfDNA CpG methylation levels

Slightly higher mapping efficiencies were achieved for the *cfNOMe* datasets from blood-derived cfDNA (81.5% ± 1.1%) compared to datasets generated from bisulfite-treated cfDNA (77.6% ± 0.1%, *p* = 1.2E−08). *cfNOMe* datasets from urine-derived cfDNA showed variable mapping efficiencies (72.4% ± 5.6%; Additional file 1: Fig. S3). Pyrosequencing assays across several genomic loci in *SLC34A1*, *UMOD*, and *CTXN3* of blood- and urine-derived cfDNA from individuals K16, K17, and P25 showed a low to moderate inter-replicate methylation variability for ECC (average SD in blood-derived cfDNA of 3.5%, range 1.4–6.9%; average SD in urine-derived cfDNA of 4.5%, range 1.1–13.0%). Further, we observed a high correlation between results from pyrosequencing assays and NGS analyses (*R* = 0.93, *p* = 2.6E−15; Additional file 1: Fig. S10). NGS genome-wide CpG methylation levels were concordant between technical replicates (average SD of 0.4%, range 0–1.4%) and between samples drawn from one control individual at different time points (blood drawing from healthy individual K17 at noon and 9:30 pm on subsequent days and urine collections from K17 at 8:00 am, noon, and 9:30 pm on subsequent days; SD of 0.1% and 1.8% for blood- and urine-derived cfDNA, respectively; Additional file 1: Fig. S6), demonstrating high robustness. Pyrosequencing the same PyroMark PCR product repeatedly also gave identical results, implicating the PyroMark PCR itself as the source of the methylation variability in our pyrosequencing assays. The *cfNOMe* libraries of individuals in the second recruitment cohort showed an average lower proportion of unconverted (=methylated) CpGs compared to both the first recruitment cohort and the bisulfite-treated samples of the second cohort. The methylated positive control DNA also showed a lower percentage of methylation in the enzymatic treatment vs. the bisulfite treatment (78.3% ± 2.4% vs. 95.5% ± 0.5%). In our experiments, the bisulfite-treated libraries compared favorably in this regard, with a higher proportion of methylated CpGs called correctly (Additional file 1: Fig. S7). Comparing *cfNOMe* and bisulfite-treated libraries, we analyzed the coverage parameters of CpG positions genome-wide. We performed a downsampling series in our ECC and BCC datasets, generating alignment files with between 1 million and 100 million aligned reads. Given equal read counts, *cfNOMe* datasets consistently covered a larger number of CpGs (48–53% more CpGs covered in *cfNOMe* datasets up to 2 × 10^7^ reads, 32% more CpGs covered at 5 × 10^7^ reads, 18% more CpGs covered at 1 × 10^8^ reads; see Additional file 1: Fig. S12 and S13).

### *cfNOMe* libraries maintain cfDNA fragmentation hallmarks and are suitable for nucleosome footprinting analyses

In order to test whether enzymatic cytosine conversion interferes with nucleosome footprinting analyses, we performed cfDNA fragmentation pattern analysis as previously described [1, 3], calculating the so-called windowed protection score (WPS_*n*_). As with unconverted cfDNA libraries, nucleosome occupancy is easily inferred from enzymatically converted cfDNA (Fig. 4a). We studied the nucleosome occupancy across the transcription start sites (TSS) of all autosomal genes in our samples, also using gene expression values from the public GTEx portal for all available non-cultured tissues. The known high degree of nucleosome organization around the TSS of transcriptionally active genes was preserved, with a strong negative aggregate WPS signal around the TSS representing the nucleosome-free region (NFR) and a strong peak for the well-phased + 1 nucleosome immediately downstream of the NFR (Fig. 4b). The pattern was less apparent in urine-derived cfDNA. This is likely due to the presence of active nucleases in the urine, the long retention period of urine in the urinary tract prior to voiding, and therefore an increased fragment degradation of urinary cfDNA in vivo [5, 33, 34] (Fig. 4c). Genes with no expression in whole blood generate no such WPS signal in blood-derived cfDNA (Additional file 1: Fig. S14). We compared the expression levels of all autosomal genes in whole blood to the nucleosome positioning signal in blood-derived enzymatically converted cfDNA around the studied genes’ TSS. We observed a robust negative correlation between gene expression rank in whole blood and the degree of nucleosomal organization. This correlation was equally strong when analyzing only the more abundant medium length cfDNA fragments of 140 bp up to 200 bp (*R* = −0.85, *p* = 1.4E−43; Fig. 4d) or the long cfDNA fragments of 200 bp up to 500 bp (*R* = −0.85, *p* = 3.2E−43; Additional file 1: Fig. S15).

We then performed a downsampling series with the largest *cfNOMe* control dataset (healthy control K16) to investigate the minimum necessary coverage for robust nucleosome occupancy studies using this assay. For aggregate analysis of nucleosome occupancy around the TSS of 1000 genes, a *cfNOMe* dataset with 200 million aligned reads still delivered a clear WPS signal. At 100 million aligned reads or less, signal noise increases. No discernable signal remains at or below 20 million aligned reads (Additional file 1: Fig. S16). It is reasonable to assume that reliable aggregate analyses of fewer positions would require larger sequencing datasets.

*cfNOMe* and bisulfite-treated libraries showed indistinguishable fragment size profiles (Additional file 1: Fig. S17 and S18). However, the correlation between the measured nucleosome positioning and gene expression levels seen in *cfNOMe* datasets was less clear in similarly sized datasets generated through bisulfite sequencing (*R* = −0.27, *p* = 0.06 for medium length fragments; Fig. 4d). We observed much weaker WPS signals in bisulfite-treated cfDNA libraries in general as the most likely explanation for this (Fig. 5a–d). Given the strong bias of bisulfite sequencing against GC-rich regions, we investigated the sequencing coverage of bisulfite-treated datasets at transcription start sites genome-wide. TSS are known to have an above average GC and CpG content [35], and a higher GC content is associated with higher rates of transcription [36]. In datasets of equal size, the TSS-specific coverage was reduced by 50% when bisulfite treatment was used (bisulfite 58.9 ± 31.6 reads; *cfNOMe* 117.6 ± 27.3 reads; *p* < 1.0E−99; Fig. 5e).

**Fig. 6:**
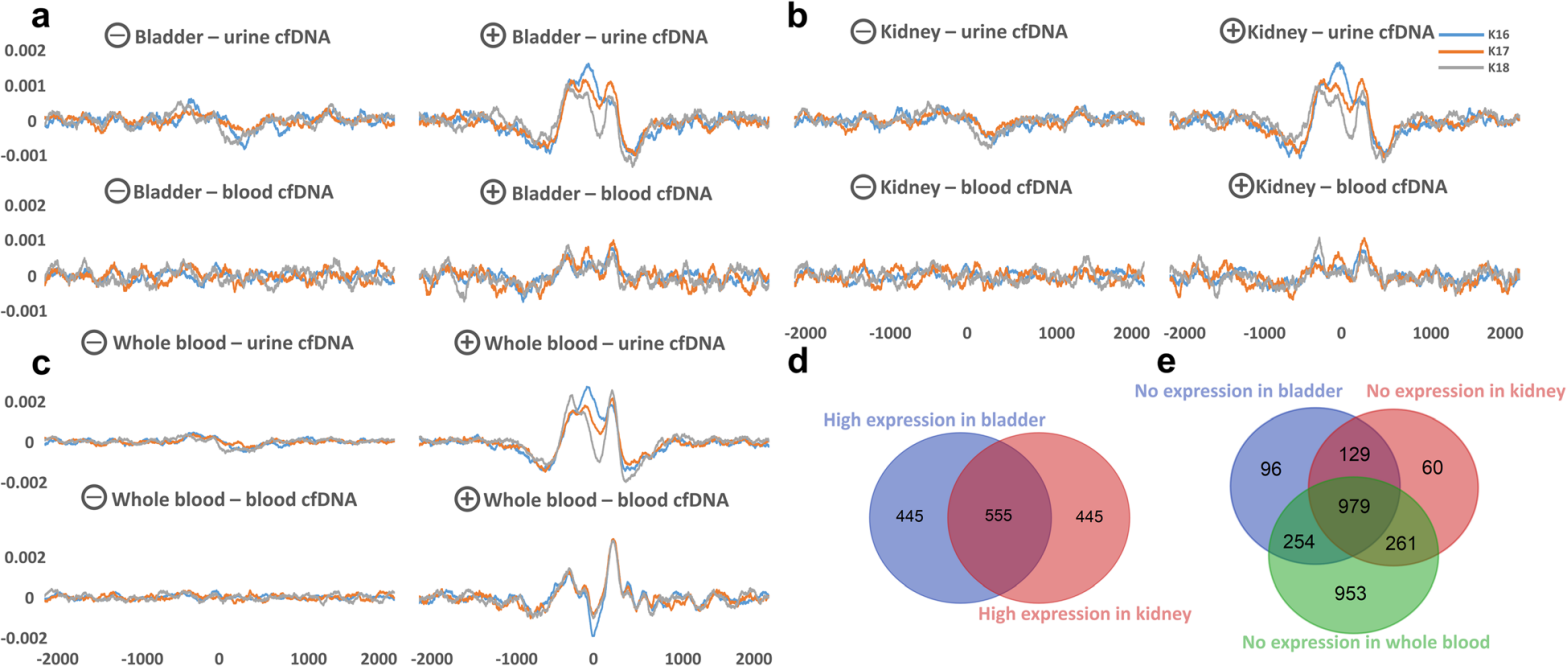
Windowed protection score with a 200-bp window size (WPS_200_) at the TSS ± 2 kb of genes with high tissue expression in the bladder and kidney, but low expression (< 0.5 transcripts per million) in whole blood. The “high expression” groups for the kidney and bladder contained genes with expression levels between 4.5 and 928.0 transcripts per million and 7.8 and 731.3 transcripts per million, respectively. For genes with the highest bladder expression (**a**) and kidney expression (**b**), a clear nucleosome positioning signal in urine-derived cfDNA, but not in blood-derived cfDNA, is visible. For comparison, genes with the highest expression in whole blood (**c**) have a WPS_200_ peak in both urine- and blood-derived cfDNA, as most of these genes are ubiquitously expressed. High-expression gene groups contain *n* = 1000 genes; zero expression gene groups contain *n* = 1458 genes for the bladder, *n* = 1429 for the kidney, and *n* = 2447 for the whole blood. **d** Overlap between genes with high kidney- and bladder-expression levels according to GTEx. About 38% of genes are shared between the two groups. **e** Overlap between genes with zero expression according to GTEx. About 36% of genes are shared between all groups. Fragments of 200–500 bp are studied in the merged datasets of the three healthy control individuals K16, K17, and K18. *X*-axes: offset from TSS in base pairs, *Y*-axes: normalized aggregate WPS_200_

Further investigating the correlation between nucleosome positioning and gene expression, we compared the nucleosomal organization around the transcription start sites of genes with high expression in the kidney and bladder between blood- and urine-derived cfDNA. Concordantly to the above results, we detected a clear nucleosomal positioning signal in urinary cfDNA, but not in blood-derived cfDNA (Fig. 6a–c). This represents an independent way to qualitatively identify large cfDNA tissue-of-origin components.

Nucleosome spacing tends to differ between genes depending on their transcriptional activity, with NFR widths being higher at the TSS of actively transcribed genes [37]. We observed significant differences of the interpeak distances (IPD [±SEM]) in the most highly expressed (311.5 ± 1.6 bp) compared to the least expressed kidney genes (294.5 ± 1.5 bp; Wilcoxon rank-sum *p* = 7.9E−12) in urine-derived cfDNA, but not when comparing highly and lowly expressed whole blood genes in blood-derived cfDNA (218.9 ± 0.6 bp vs. 213.3 ± 0.5 bp; Wilcoxon rank-sum *p* = 0.33; Additional file 1: Fig. S19).

### Deconvolution of cfDNA tissues-of-origin from *cfNOMe* data

Similarly to previously published methodology [2, 4], genome-wide methylation metrics and methylation levels at loci with a tissue-specific methylation pattern were extracted (Additional file 1: Fig. S20). We used methylation signature reference data of 25 tissues [4] (Additional file 1: Fig. S9) and performed a linear least squares-based analysis of each dataset against these references to calculate each cfDNA sample’s respective tissue composition (Additional file 1: Fig. S21 and S22).

Two patients in this study had previously received a kidney transplant for which donor DNA could be obtained: one patient (P29) from a first-degree relative and the other (P27) from an unrelated deceased donor. Patient P29 was analyzed twice: once during an episode of acute graft dysfunction, and again after improvement of graft kidney function (see Additional file 1: Table S7 for additional clinical data). Using the genotypic differences of the kidney graft, we estimated the renal cfDNA fraction in these NGS datasets. These values can be seen as the “ground truth.” Patients P29 and P27 had a kidney cfDNA fraction (± 95% CI) of 9.7% ± 0.6% and 6.0% ± 0.3% in their blood, respectively, during the acute stages of disease. After improvement of kidney function in patient P29, the renal cfDNA as calculated from genotype differences decreased markedly from 9.7% ± 0.6% to 2.9% ± 0.4%. The renal fraction in urine-derived cfDNA for patients P29 and P27 during the episode of graft rejection was calculated as 63.2% ± 1.6% and 67.7% ± 0.5%, respectively, and as 71.8% ± 1.5% in the follow-up measurement in patient P29 (Additional file 1: Fig. S4).

The renal cfDNA proportions as inferred from methylation deconvolution were overall closely correlated with the values calculated based on donor SNPs (*R* = 0.98, *p* = 6.3E−04; Additional file 1: Fig. S23a), although the deconvolution approach did not show a corresponding decrease in the renal cfDNA of patient P29 after recovery. Additionally, the deconvolution-inferred values for blood-derived renal cfDNA were significantly lower in healthy controls than in patients during acute kidney injury or kidney graft dysfunction (*p* = 0.002; Additional file 1: Fig. S23b).

This kidney graft-dependent validation of renal cfDNA was not possible in patient P25 suffering from acute kidney injury in the absence of a renal allograft. However, methylation deconvolution also detected elevated levels of renal cfDNA in the blood of this individual.

In the replication cohort, we notably detected the highest proportion of pancreatic and hepatic cfDNA in the blood of patient P30, who suffered from acute necrotizing pancreatitis and had elevated liver enzymes at the time of sample collection (Additional file 1: Fig. S24). Patient P31, suffering from polycystic kidney disease, showed elevated levels of renal cfDNA in the blood (3.2% vs. 1.0% ± 0.6% in six healthy controls). Patient P32 with primary hyperoxaluria type 1 (PH1) and more than 5 years on chronic hemodialysis after kidney graft loss also displayed (less markedly) increased renal cfDNA levels in her blood (2.0%); however, we were unable to detect any additional perturbations in cfDNA composition related to her other main clinical problem of PH1-related bone disease. It should be noted that no methylation reference for the bone was included in the used tissue reference set. An increase in bone-associated cfDNA would therefore not be recognizable to the deconvolution algorithm.

Our deconvolution results for all 25 studied tissues were highly correlated between technical replicates, as well as between the two blood samples and three urine samples collected from healthy individual K17 at different time points (Additional file 1: Fig. S25). To fully assess the variability of cellular turnover in healthy individuals, however, a larger set of healthy control individuals would be needed.

## Discussion

Using a non-disruptive enzymatic method of cytosine conversion, we are able to generate high-fidelity methylome datasets from cfDNA. We performed analyses of two independent epigenetic modifications, cytosine methylation and nucleosome occupancy, with the help of a custom bioinformatic analysis toolkit. Together, we term this method “cell-free DNA-based Nucleosome Occupancy and Methylation profiling” (*cfNOMe*).

Unlike genotype-based studies of cell-free DNA, whose diagnostic usefulness is currently limited to cancer [8], transplantation [10], and pregnancy [9], epigenetic profiling of cfDNA promises almost universal applicability if a number of methodological challenges can be overcome.

In our experiments, bisulfite conversion performed better in terms of cytosine conversion efficiency. We observed over-conversion in one of our two enzymatically converted datasets. A new version of the enzymatic conversion kit has since been released, promising improved conversion robustness and reliability. Still, in terms of uniformity of coverage, enzymatic conversion delivered much improved results compared to bisulfite treatment. However, we did not perform a detailed or systematic comparison of the methylation conversion rate between bisulfite and enzymatic treatment in this study, meaning that these observations cannot be generalized. Methylation analyses using enzymatic conversion, including the methylation-based deconvolution of cfDNA tissues-of-origin, generated reproducible and reliable results between replicates. By including two kidney transplant recipients in our study, we were able to compare the deconvolution results directly with the established method of calculating kidney graft cfDNA fractions using genotype differences between donor and recipient. While there was generally good agreement between the two approaches, small discrepancies remained. An imprecision of methylation deconvolution on the order of several percentage points has been observed consistently in prior studies [2, 4, 5] and remains to be overcome. In patients with acute renal dysfunction from acute kidney injury and graft rejection, we observed higher proportions of renal cfDNA in blood using methylation deconvolution, in line with earlier results from genotype-based allograft dysfunction studies [10]. Higher fractions of renal cfDNA were also seen in two out of three patients with chronic kidney disease, as well as high levels of pancreatic and hepatic cfDNA in a patient on chronic peritoneal dialysis for atypical hemolytic uremic syndrome-associated end-stage renal disease complicated by acute necrotizing pancreatitis and elevated liver enzymes. Using a diverse bisulfite-converted reference dataset of 25 tissues [4] in our deconvolution approach, we observed a high degree of inter-individual variability in the calculated tissue composition of cfDNA. Particularly, hepatocyte- and adipocyte-derived cfDNA was detected by the deconvolution algorithm in several healthy controls and patients. While levels of hepatic cfDNA ≥ 1% in healthy controls and even ≥ 50% in sepsis and hepatopathy patients have reliably been observed in other studies [2, 4], the adipocyte contribution had either not been measured or been measured to be negligible. As such, it is unclear whether these proof-of-principle deconvolution results, measuring enzymatically converted samples against bisulfite-converted references, are fully accurate. We also calculated significant proportions of prostate-derived cfDNA in the urine of patients and controls, irrespective of the individual’s sex. As the cladogram in Additional file 1: Fig. S9 shows, the prostate methylation reference is very similar to the bladder and kidney references, likely increasing the risk for this kind of misclassification. A previous study on the composition of urinary cfDNA by Cheng et al. [5] did not contain a prostate or bladder tissue reference, but an urothelium reference. The authors of this study measured highly variable levels of urothelium-derived cfDNA between 0 and 64% in the urine of kidney transplant recipients.

Collectively, these observations highlight important limitations of this study and the need for future research to (1) generate enzymatically converted reference datasets for use with *cfNOMe* or other ECC-based cfDNA deconvolution studies and (2) generate comprehensive cell type-specific methylation references. This will, in our estimation, reduce the risk of misclassification between heterogeneous tissues with overlapping cellular compositions and further improve the resolution and sensitivity of future assays required for diagnostic purposes.

Additionally, the expected accuracy of NGS-based methylation analyses is a function of the sequence coverage at the position of interest. We therefore envision that targeted enrichment approaches in conjunction with enzymatic cytosine conversion could enable the inexpensive generation of datasets with high coverage depths for even more reliable cfDNA methylation studies.

We show that the enzymatic cytosine conversion approach does not interfere with even highly sensitive fragmentation analyses to study nucleosome occupancy, as the cfDNA fragmentation information is fully preserved. While nucleosome occupancy studies are also possible on bisulfite-converted sequence libraries at a lower resolution, we observed significantly weaker nucleosome footprinting signals at similar sequencing depths in the same samples. Using the *cfNOMe* approach, nucleosome occupancy studies are turned from an expensive add-on test into an inexpensive in silico analysis step, bringing them closer to a broader clinical application. Consequently, one major takeaway of this study is whenever cfDNA methylome data is being generated in this way, investigators should also take the opportunity to look at its fragmentation patterns for additional insight, e.g., as was recently demonstrated by Ulz and colleagues in a cancer context [15].

Our findings also indicate that such nucleosome occupancy studies are more problematic in urine-derived cfDNA, with the substantial in vivo degradation of cfDNA fragments [5] obscuring the underlying nucleosome-associated fragmentation pattern signals. We also did not observe a clinically meaningful difference in the urinary renal cfDNA fraction of a patient with acute kidney graft rejection before and after improvement of renal function, in good agreement with previous reports of a very large variability in urinary cfDNA composition in states of both health and disease [5]. For these reasons, we currently regard blood-derived cfDNA deconvolution and nucleosome occupancy studies as overall more promising in terms of diagnostic usefulness and broad applicability.

## Conclusions

In conclusion, the *cfNOMe* assay for epigenetic analysis of cfDNA is a novel two-in-one workflow that enables more comprehensive, efficient, and affordable studies of the cfDNA-associated epigenetic landscape. Fragmentation analyses can be done on converted datasets with a low amount of effort. We present proof-of-principle data for the utility of methylation, as well as nucleosome occupancy signatures, in identifying and quantifying cfDNA tissues-of-origin. Future research will need to expand on the synergies of methylation and nucleosome occupancy profiling in the clinical context.

## Supplementary information

**Additional file 1: Figure S1**. Basic principle of enzymatic cytosine conversion. **Figure S2**. Coverage by GC content of sequenced DNA. **Figure S3**. Comparison of mapping efficiency by method and input material. **Figure S4**. Donor SNP allele fraction in graft, patient blood and patient urine for P27 and P29. **Figure S5**. Methylation bias by read position. **Figure S6**. Global methylation levels in replicates for K16, K17, K18, P25, P27 and P29. **Figure S7**. Global methylation levels in K19, K20, K21 (Bisulfite and cfNOMe) and P30, P31 and P32. **Figure S8**. CpG and non-CpG methylation levels on chromosome 2. **Figure S9**. Clustermap of the methylation profiles of 25 reference tissues. **Figure S10**. Correlation between pyrosequencing and NGS methylation calls in enzymatic conversion. **Figure S11**. Cohort and experimental overview. **Figure S12**. CpG positions covered ≥1x in cfNOMe vs. Bisulfite sequencing. **Figure S13**. Coverage depth over CpG positions in cfNOMe vs. Bisulfite sequencing. **Figure S14**. Normalized WPS_120_ around the transcription start site (TSS) of expressed vs. non-expressed genes in whole blood. **Figure S15**. Correlation between the nucleosome positioning signal (NPS) and the level of gene expression, 200-500 bp cfDNA fragments. **Figure S16**. Downsampling series of nucleosome positioning signal as a function of dataset size. **Figure S17**. Blood-derived cfDNA fragment length histograms. **Figure S18**. Urine-derived cfDNA fragment length histograms. **Figure S19**. Violin plot of the interpeak distances in the WPS signal around high- and low-expression genes. **Figure S20**. Methylation profiles for all samples at 7890 informative differentially methylated CpGs. **Figure S21**. Methylation deconvolution results for blood-derived cfDNA samples. **Figure S22**. Methylation deconvolution results for urine-derived cfDNA samples. **Figure S23**. Deconvolution-calculated renal cfDNA fractions in patients vs. controls. **Figure S24**. Deconvolution-calculated cfDNA fractions with liver and pancreas tissue-of-origin in all samples. **Figure S25**. Pair-wise comparison of deconvolution results in replicates of the same sample. **Table S1**. Library preparation steps and DNA amounts in urine samples. **Table S2**. Library preparation steps and DNA amounts in blood samples, discovery cohort. **Table S3**. Library preparation steps and DNA amounts in blood samples, replication cohort. **Table S4**. Reaction composition using PyroMark PCR Master Mix. **Table S5**. PCR protocol for PyroMark PCR reaction. **Table S6**. Primer sequences and reaction conditions for loci of interest. **Table S7**. Sequencing and associated clinical data for each sample in this study, discovery cohort. **Table S8**. Sequencing and associated clinical data for each sample in this study, replication cohort.

## Data Availability

Raw sequencing data has been deposited in the European Genome-phenome Archive (Accession ID EGAS00001004370, https://ega-archive.org/studies/EGAS00001004370) [30]. The data used for stratification by gene expression in various tissues were obtained from the GTEx Portal on 21 March 2019, specifically as part of the GTEx Analysis v7 “Mean gene-level TPN by tissue”, dated 15 January 2016 (dbGaP Study Accession phs000424.v7.p2) [38]. Custom computer code used to generate the data in this study, as well as demo data and usage instructions are available on github (see below). Further code declaration: Project name: cfNOMe toolkit [39] Project home page: https://github.com/FlorianErger/cfNOMe Archived version: 1.0 Operating system(s): Linux Programming language: Python 3.7, GNU bash Other requirements: GNU parallel, samtools, Python scipy, Python numpy, bismark, bowtie2, bedmap, sort-bed License: the *cfNOMe* toolkit is released under the GNU GPLv3 license Any restrictions to use by non-academics: none
