# Supplementary material for: cfNOMe — A single assay for comprehensive epigenetic analyses of cell-free DNA
Source: Genome Med. 2020 Jun 24;12:54. doi: 10.1186/s13073-020-00750-5 (PMC7315486; doi:10.1186/s13073-020-00750-5)
Supplement: Supplementary file 1 — Additional file 1: Figure S1. Basic principle of enzymatic cytosine conversion. Figure S2. Coverage by GC content of sequenced DNA. Figure S3. Comparison of mapping efficiency by method and input material. Figure S4. Donor SNP allele fraction in graft, patient blood and patient urine for P27 and P29. Figure S5. Methylation bias by read position. Figure S6. Global methylation levels in replicates for K16, K17, K18, P25, P27 and P29. Figure S7. Global methylation levels in K19, K20, K21 (Bisulfite and cfNOMe) and P30, P31 and P32. Figure S8. CpG and non-CpG methylation levels on chromosome 2. Figure S9. Clustermap of the methylation profiles of 25 reference tissues. Figure S10. Correlation between pyrosequencing and NGS methylation calls in enzymatic conversion. Figure S11. Cohort and experimental overview. Figure S12. CpG positions covered ≥1x in cfNOMe vs. Bisulfite sequencing. Figure S13. Coverage depth over CpG positions in cfNOMe vs. Bisulfite sequencing. Figure S14. Normalized WPS120 around the transcription start site (TSS) of expressed vs. non-expressed genes in whole blood. Figure S15. Correlation between the nucleosome positioning signal (NPS) and the level of gene expression, 200-500 bp cfDNA fragments. Figure S16. Downsampling series of nucleosome positioning signal as a function of dataset size. Figure S17. Blood-derived cfDNA fragment length histograms. Figure S18. Urine-derived cfDNA fragment length histograms. Figure S19. Violin plot of the interpeak distances in the WPS signal around high- and low-expression genes. Figure S20. Methylation profiles for all samples at 7890 informative differentially methylated CpGs. Figure S21. Methylation deconvolution results for blood-derived cfDNA samples. Figure S22. Methylation deconvolution results for urine-derived cfDNA samples. Figure S23. Deconvolution-calculated renal cfDNA fractions in patients vs. controls. Figure S24. Deconvolution-calculated cfDNA fractions with liver and pancreas tissue-of-origin in all [file 13073_2020_750_MOESM1_ESM.docx]

**Additional file 1: Supplementary materials**


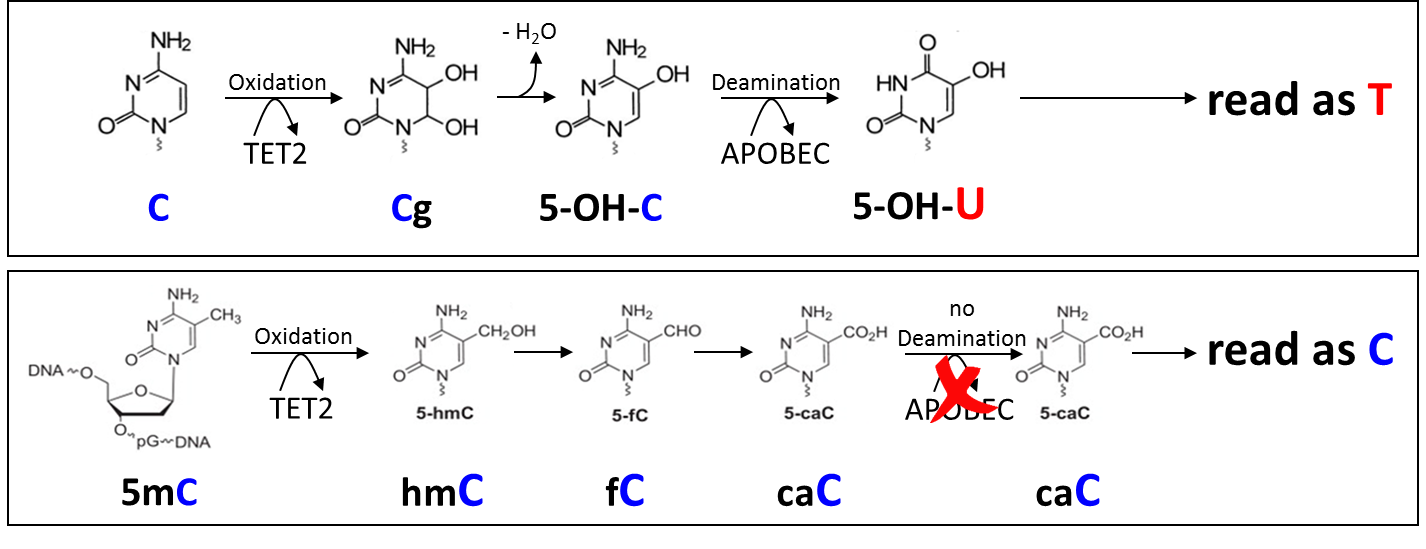


Fig. S1: Basic principle of the used enzymatic cytosine conversion approach: Enzymatic modification by TET2 and APOBEC of non-methylated cytosines allows methylation-sensitive sequencing. The TET2 enzyme and an oxidation enhancer reagent selectively and non-destructively convert both 5mC and 5hmC into oxidized derivatives that are no longer substrates of the APOBEC enzyme. APOBEC subsequently deaminates non-5mC and non-5hmC cytosines. Upon sequencing, 5mC and 5hmC are then read as cytosines, whereas the APOBEC-deaminated cytosines are read as thymines. C: cytosine, Cg: cytosine glycol, 5-OH-C: 5-hydroxycytosine, 5-OH-U: 5-hydroxyuracil, T: thymine, 5mC: 5-methylcytosine, hmC: 5-hydroxymethylcytosine, fC: formylcytosine, caC; carboxycytosine.


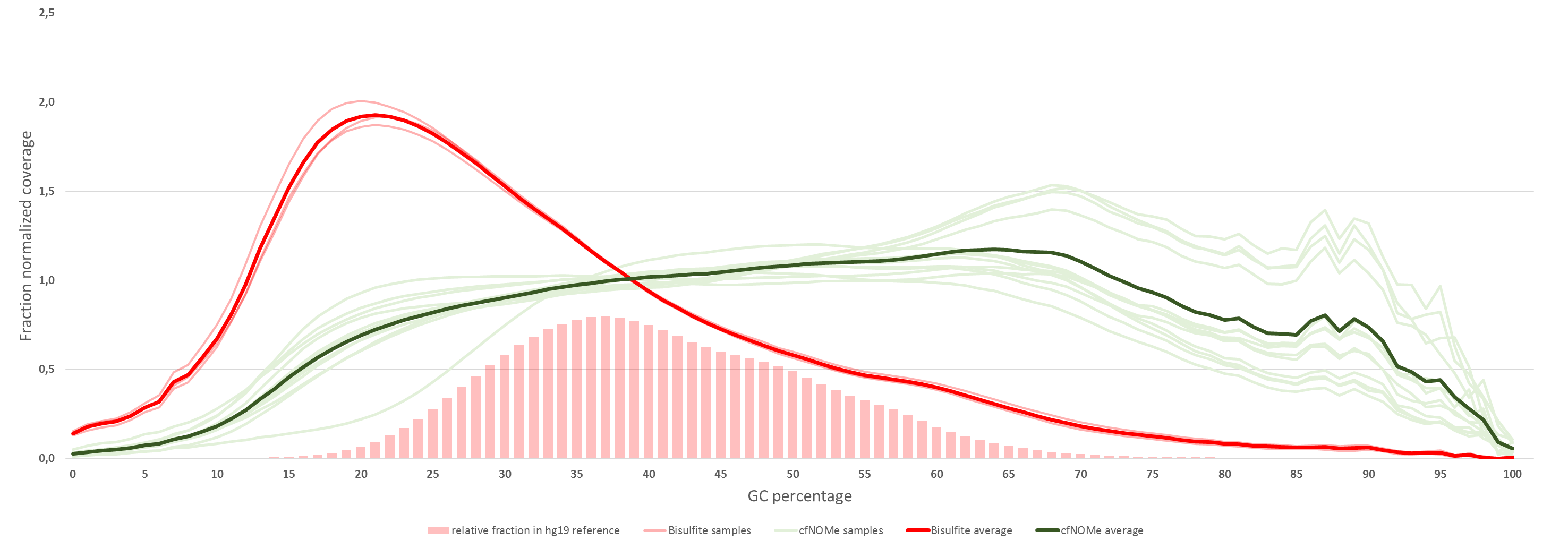


Fig. S2: Normalized coverage over genomic regions plotted by GC content. Enzymatic methylation sequencing (n=13 samples) achieves more balanced coverage than Bisulfite sequencing (n=3 samples) by more proportionally representing GC-rich regions in the studied libraries. The CollectGcBiasMetrics tool of the Picard package (http://broadinstitute.github.io/picard) was used on each merged library and the subsequent results averaged. All libraries were studied.


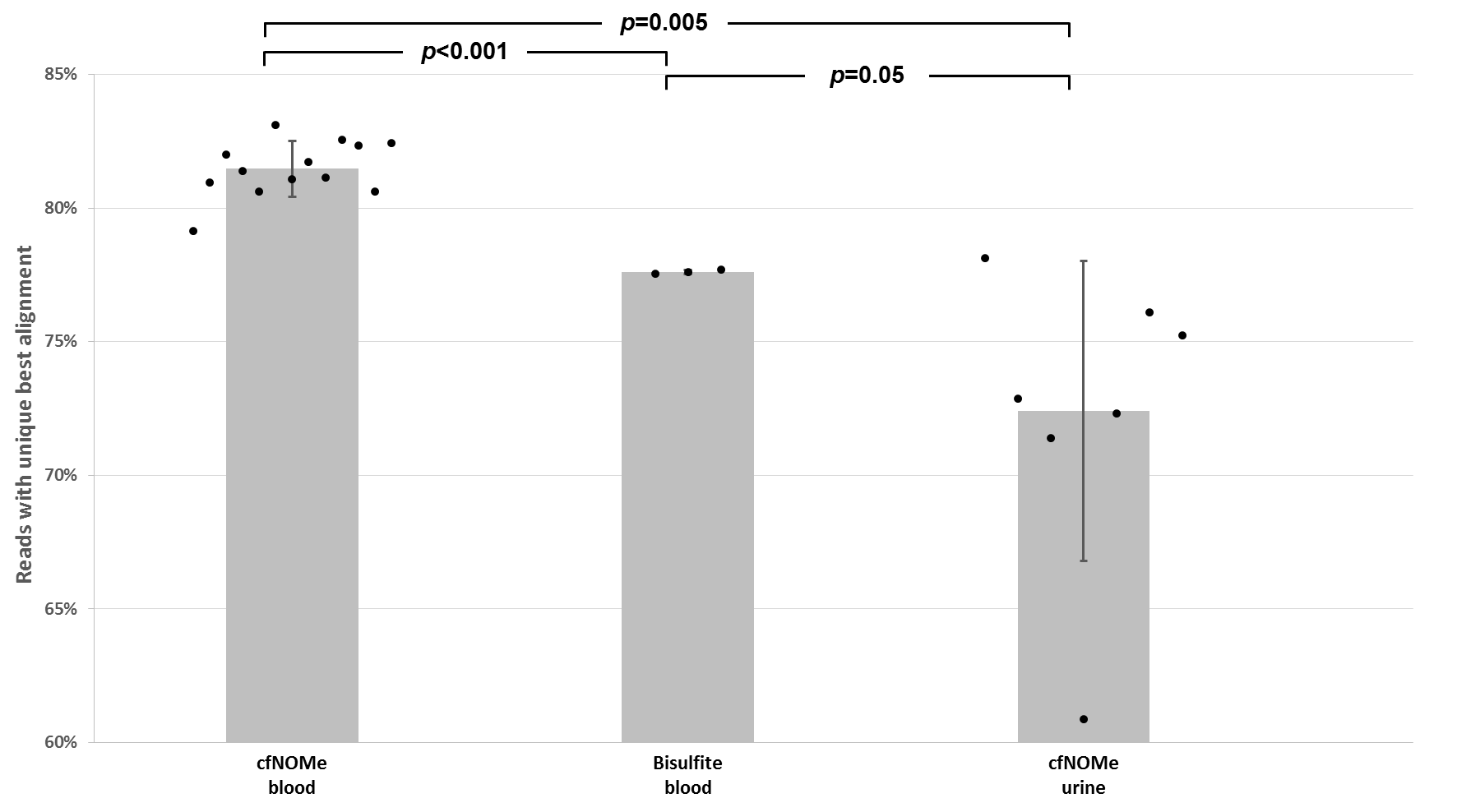
 Fig. S3: Mapping efficiency of methylation-sensitive alignment for all analysed samples (merged libraries). The mapping quality was highest for blood-derived cfDNA after enzymatic cytosine conversion. Urine-derived, enzymatically converted cfDNA libraries showed a highly variable mapping efficiency. Error bars represent SD.


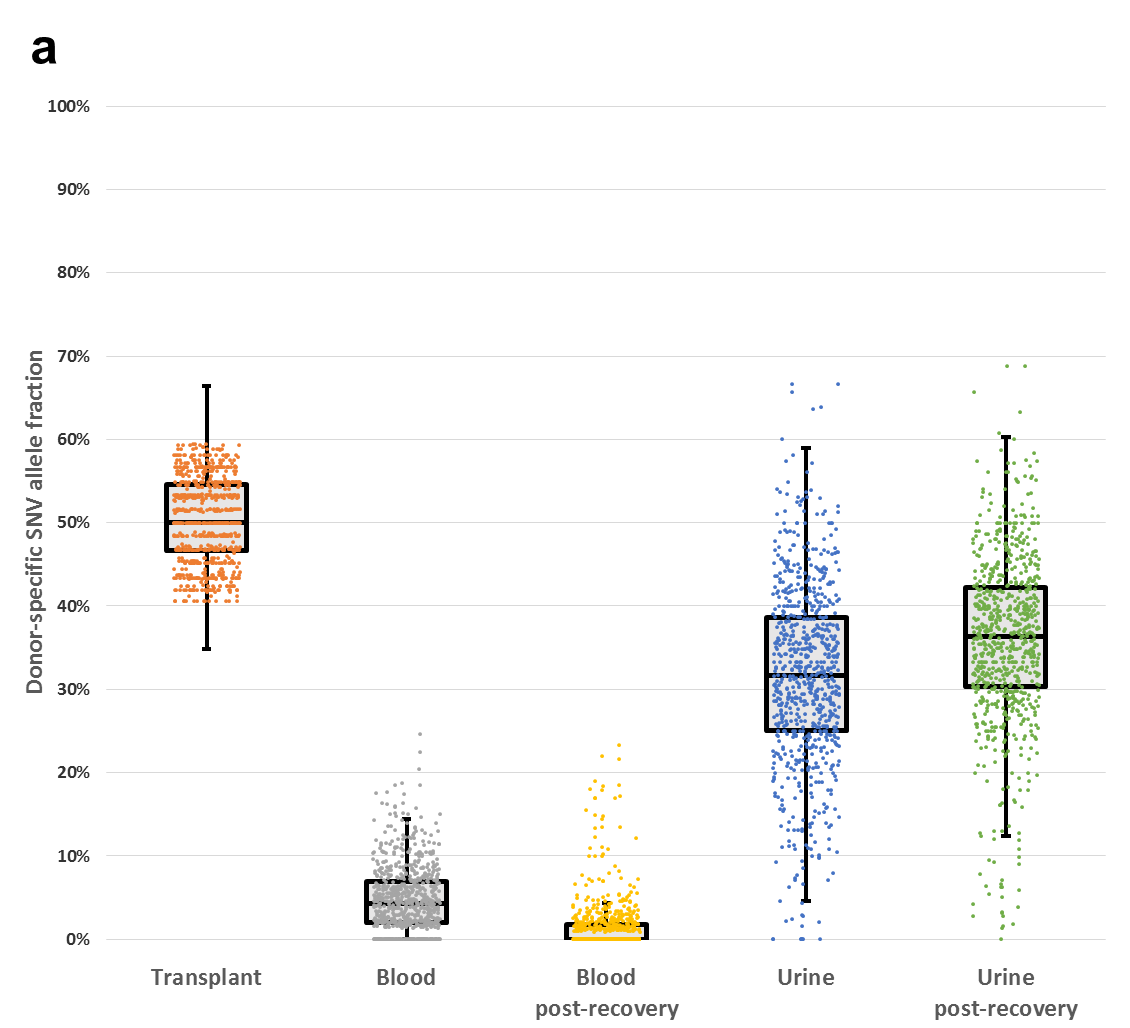

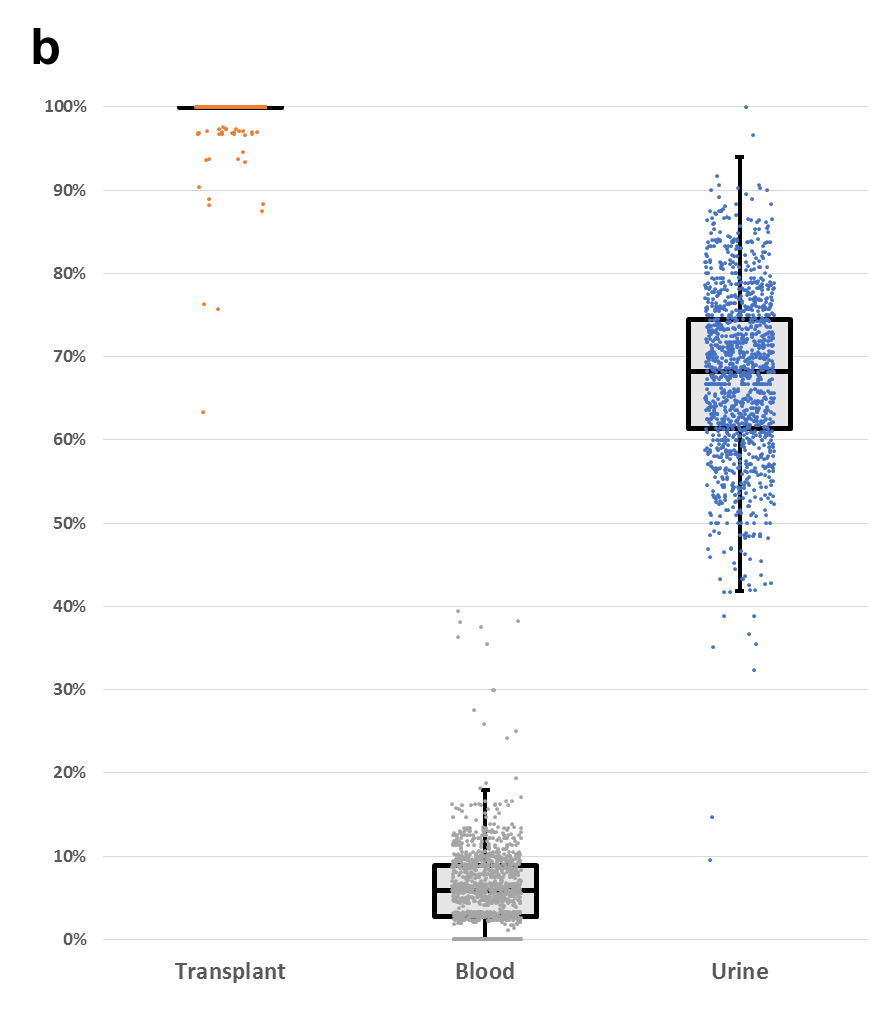


Fig. S4: Donor SNP allele fraction in graft, patient blood and patient urine. **a**, Patient P29 with a first-degree relative donor kidney transplant. Since only heterozygous donor SNPs (n=776) could be analysed, allele fractions were doubled to arrive at the percentage of graft (i.e. renal) cfDNA. **b**, Patient P27 with unrelated donor kidney transplant (n=1,326 donor SNPs). Dots represent allele fractions of individual SNPs. Horizontal lines in boxplots represent Q1, median and Q3. Whiskers represent 1.5x interquartile range.


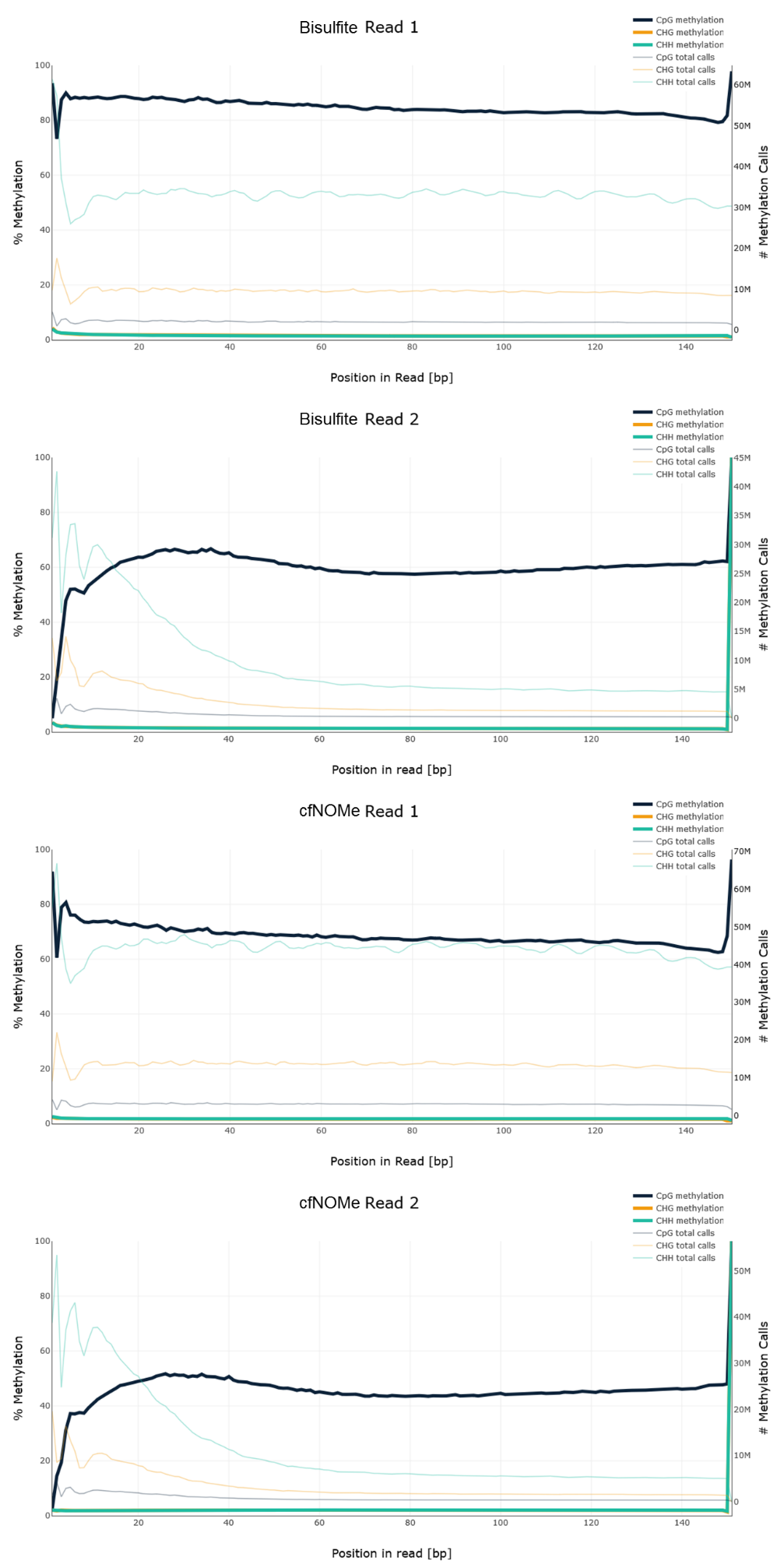
Fig. S5: Methylation bias by read position in healthy control K19. For both methods, a strong methylation bias is observed at the ends of both reads. The drop-off in methylation calls over the length of read2 is caused by the Bismark tools default --no_overlap behaviour, which discards methylation calls in read2, if the same cytosine was already assessed in read1 of the same fragment. This occurs when fragments are short and both reads overlap in the middle of the insert.


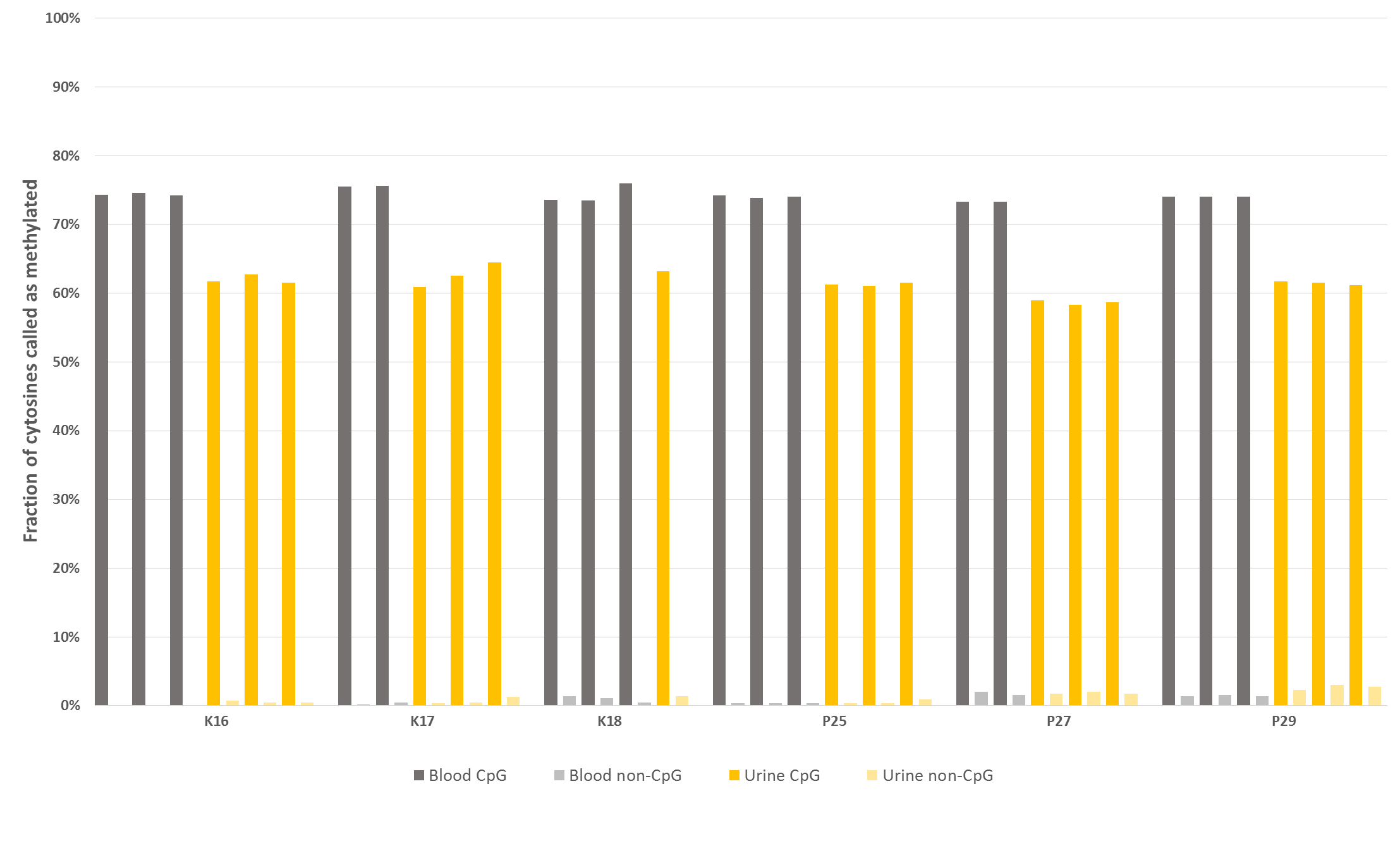
 Fig. S6: Discovery cohort: Global methylation comparison between technical and biological (K17) replicates (see Table S7). Close agreement between genome-wide CpG methylation levels in sample replicates using the *cfNOMe* assay and NGS sequencing. Both CpG and non-CpG context is shown. For the control sample K17 and patient sample P27, only two replicates of the blood-derived cfDNA were compared. The urine-derived cfDNA of K18 was sequenced from a single library. K16, K17, K18: healthy controls. P25, P27, P29: acute kidney injury/kidney graft dysfunction patients.


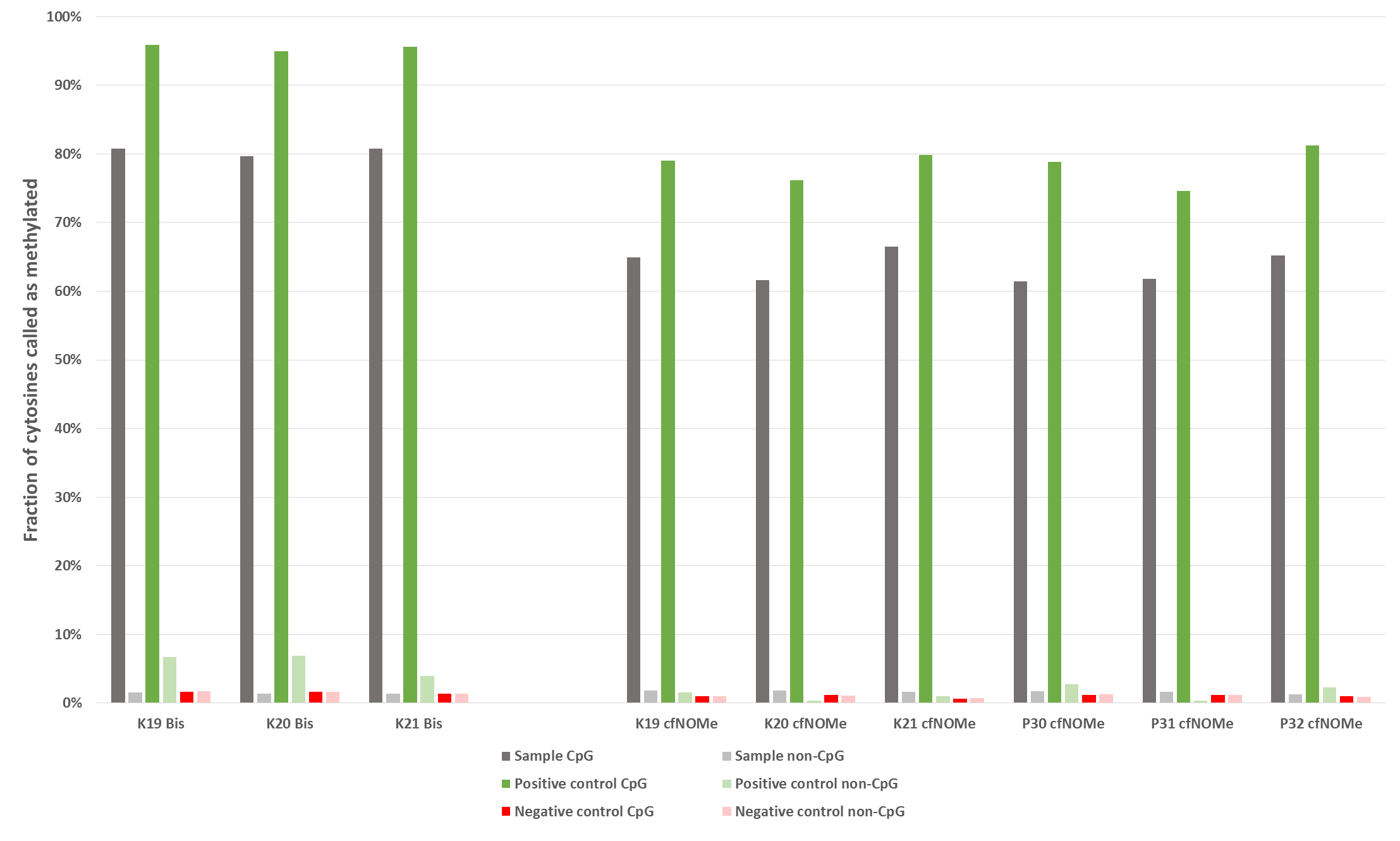


Fig. S7: Replication cohort: Global methylation comparison between different conversion methods. All shown samples were processed and sequenced together. The measured methylation levels in the sample and positive control was lower in cfNOMe samples, whereas methylation calls in a non-CpG context were increased in the Bisulfite-converted positive controls.


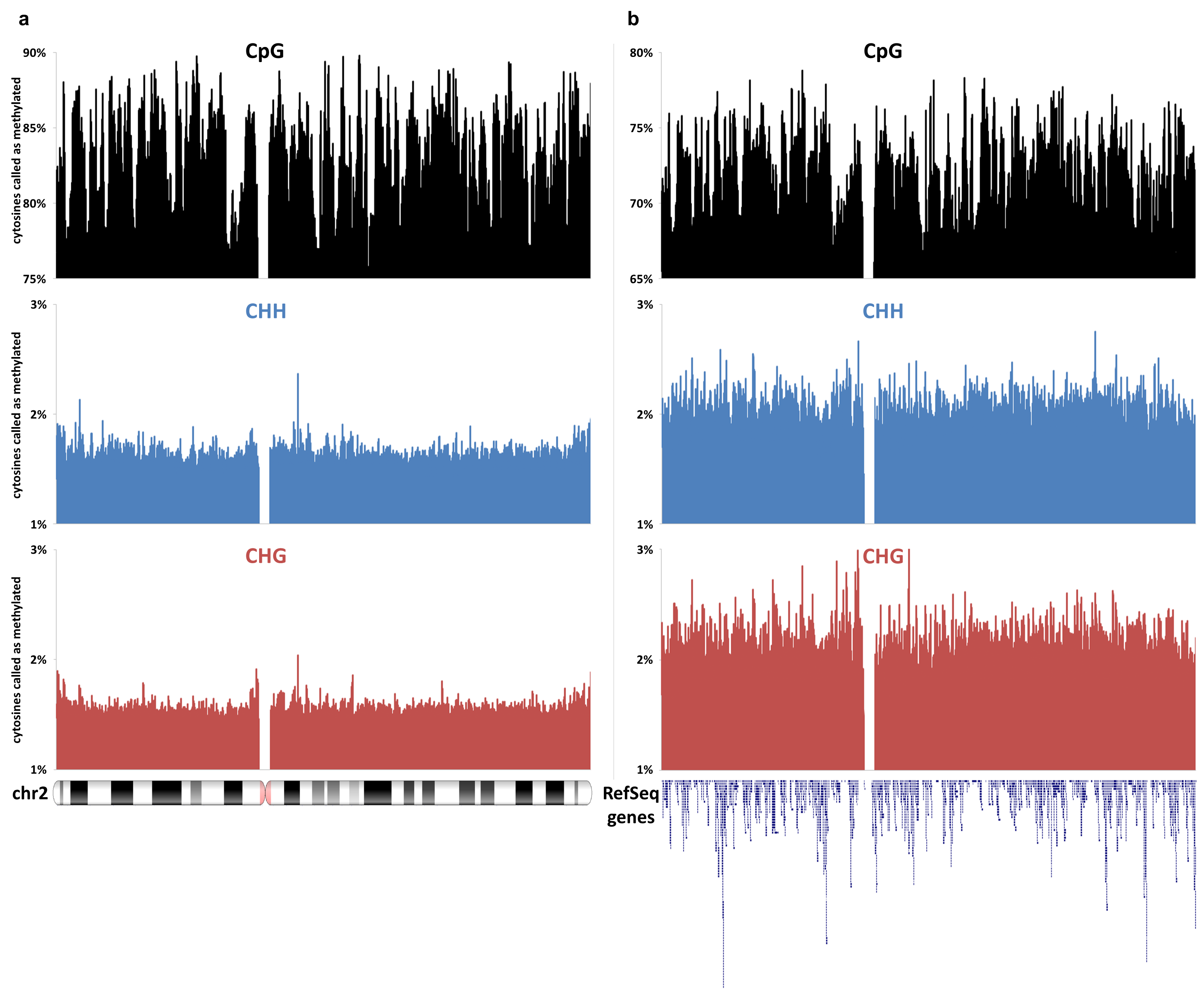


Fig. S8: CpG and non-CpG methylation levels by genomic position on chromosome 2. Both Bisulfite conversion (**a**) and enzymatic conversion (**b**) concordantly reveal the complex landscape of CpG-methylation along the length of the chromosome. The context-specific methylation calls of control sample K19 were calculated for 1000bp windows and a running average was plotted along the length of chromosome 2. No methylation calls were made in the centromeric region due to missing alignment.


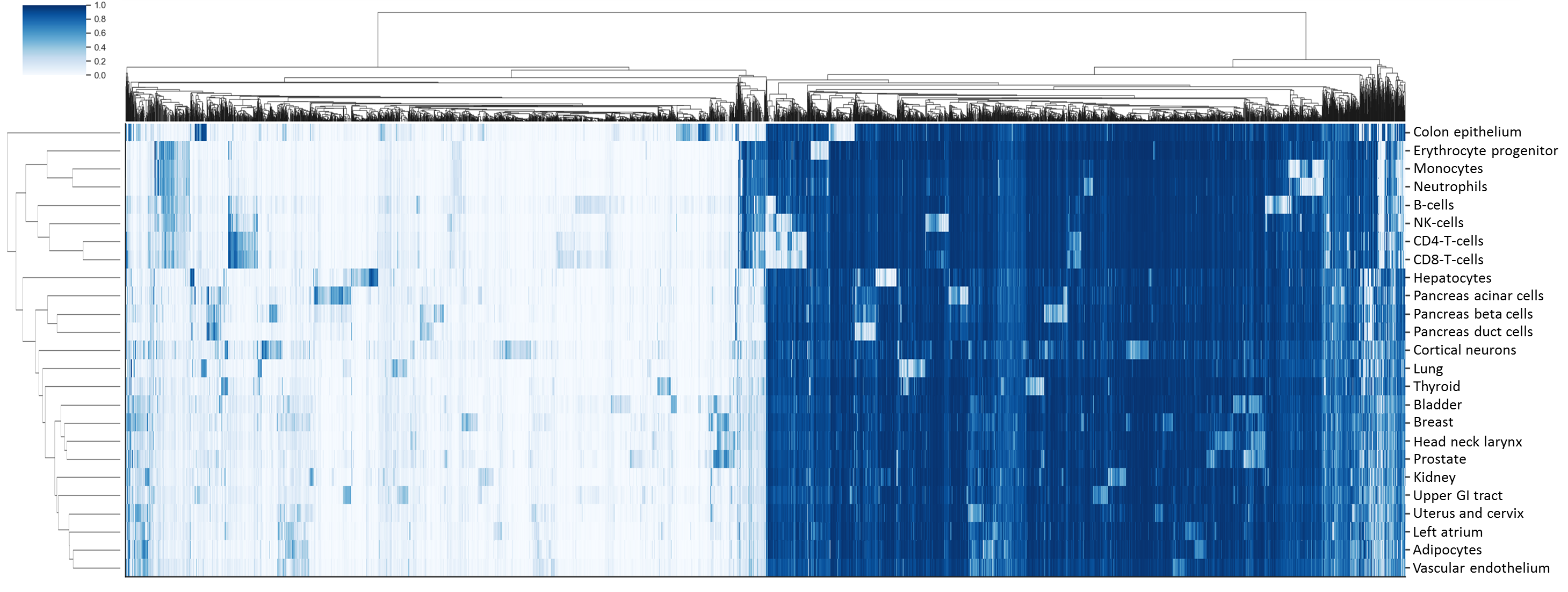


Fig. S9: Clustermap of the methylation profiles of 25 reference tissues and 7890 tissue-specific CpG loci from Moss and colleagues[1]. Biologically similar tissues (e.g. lymphoid and myeloid cells, different pancreatic cell subtypes) cluster together as expected. Light blue: low methylation grade, dark blue: high methylation grade.


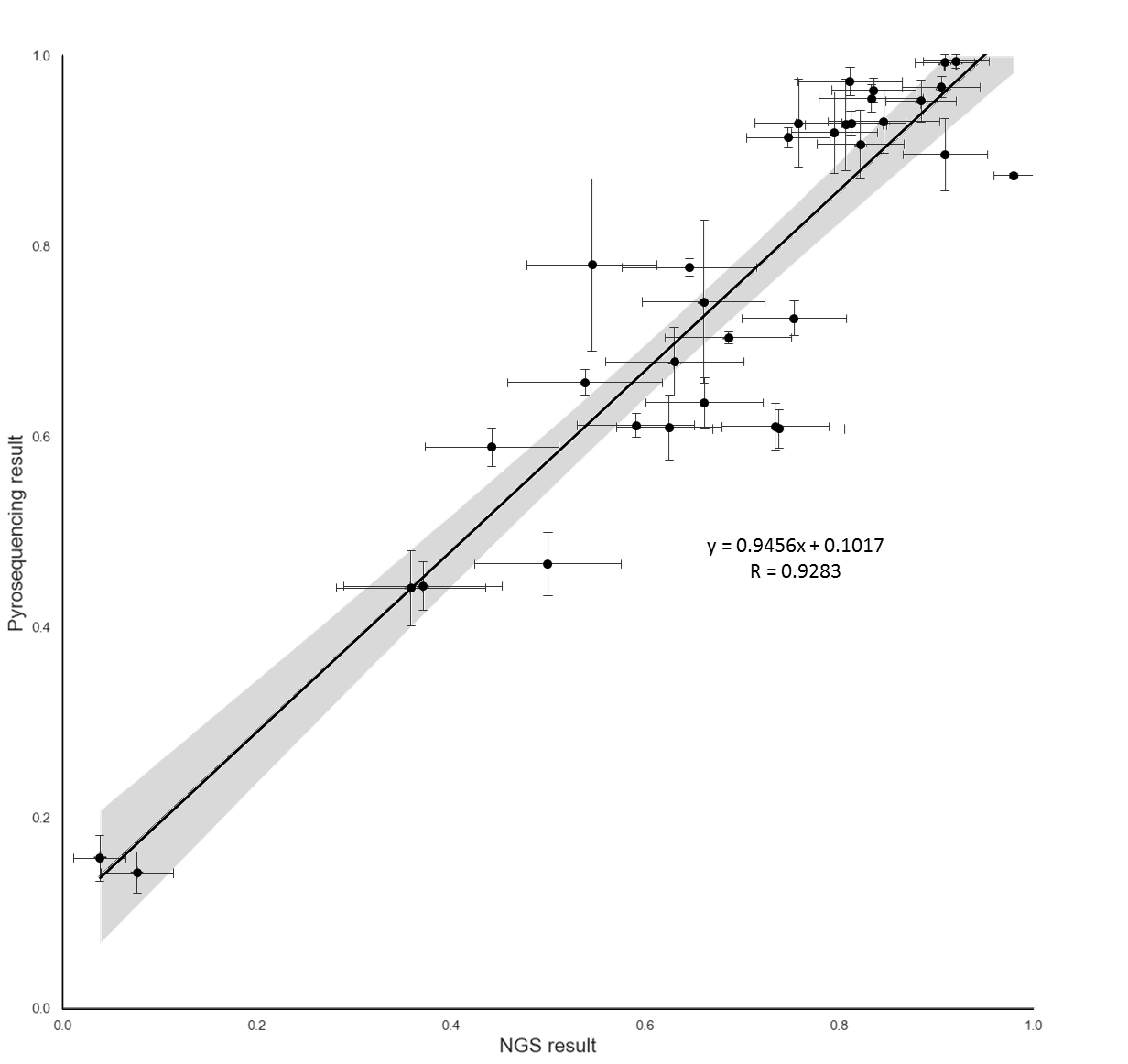
Fig. S10: Agreement of pyrosequencing and NGS cytosine methylation analysis results (n=34 measurement pairs in merged blood and urine samples of healthy controls K16 and K17, as well as of patient P25; six loci of interest in *SLC34A1*, *UMOD* and *CTXN3* were studied in all samples, except for the P25 blood sample, for which four loci of interest could be pyrosequenced). There is a close correlation between CpG methylation levels as measured by pyrosequencing and as calculated from NGS data. Pyrosequencing done in triplicate. Error bars represent SEM. Shaded area represents the 95% confidence band of the regression model.


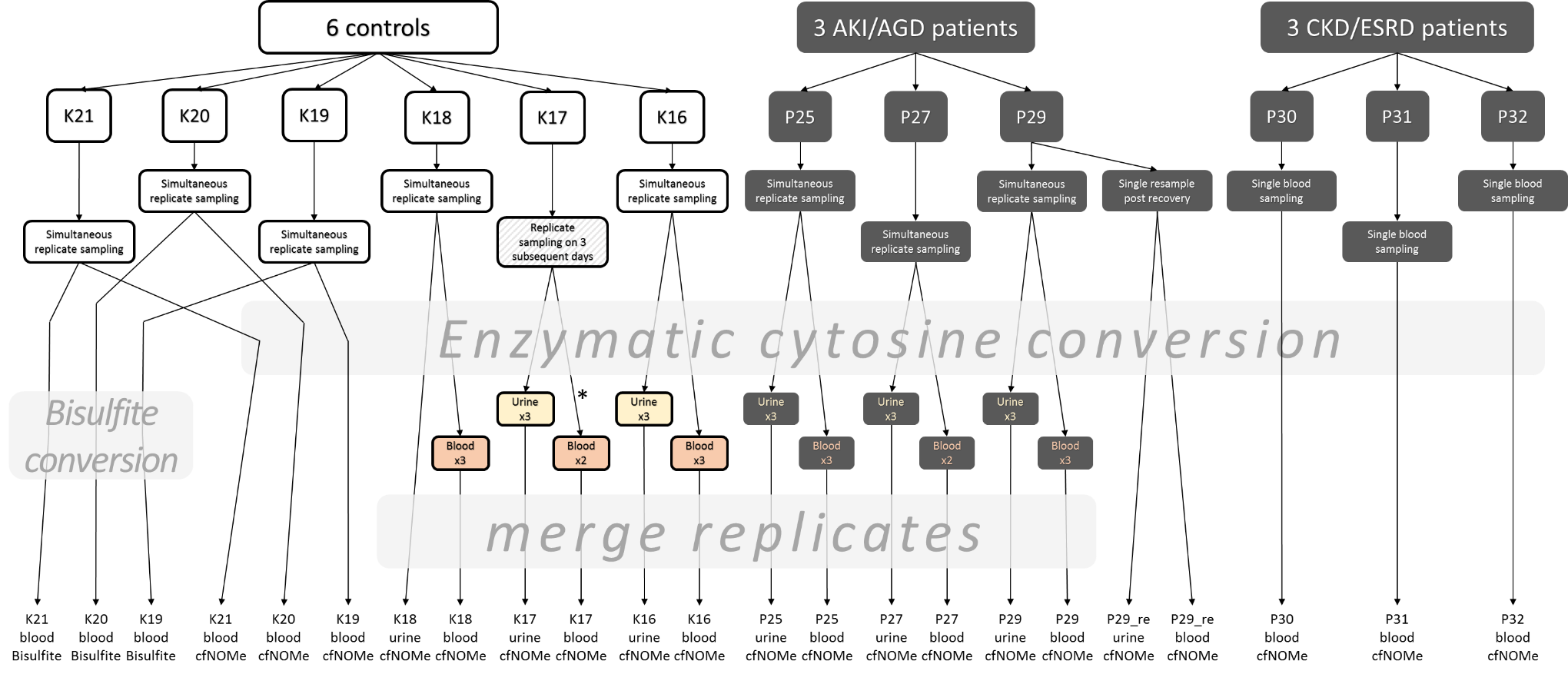


Fig. S11: Cohort and experimental overview. Six controls (K16-K21) were analysed. Three samples from healthy controls (K19-K21) were split prior to conversion and then processed with Bisulfite conversion and enzymatic conversion in parallel. Blood/urine availability permitting, samples were drawn in triplicate, processed separately and then analysed both individually and after merging. One subgroup of three patients (P25, P27, P29) with acute kidney injury or acute kidney graft dysfunction (AKI/AGD), as well as a group of three patients with progressive chronic kidney disease (P31, polycystic kidneys) or endstage renal disease (P30, status post atypical hemolytic uremic syndrome with acute pancreatitis and P32, primary hyperoxaluria type 1 with bone oxalosis) without and with extrarenal manifestations of tissue dysfunction were selected. Urine was analysed in the discovery cohort (K16, K17, K18, P25, P27, P29) only.

*: one blood replicate of patient K17 was hemolytic. As we observed high levels of contamination with genomic DNA, this sample was not deep-sequenced.


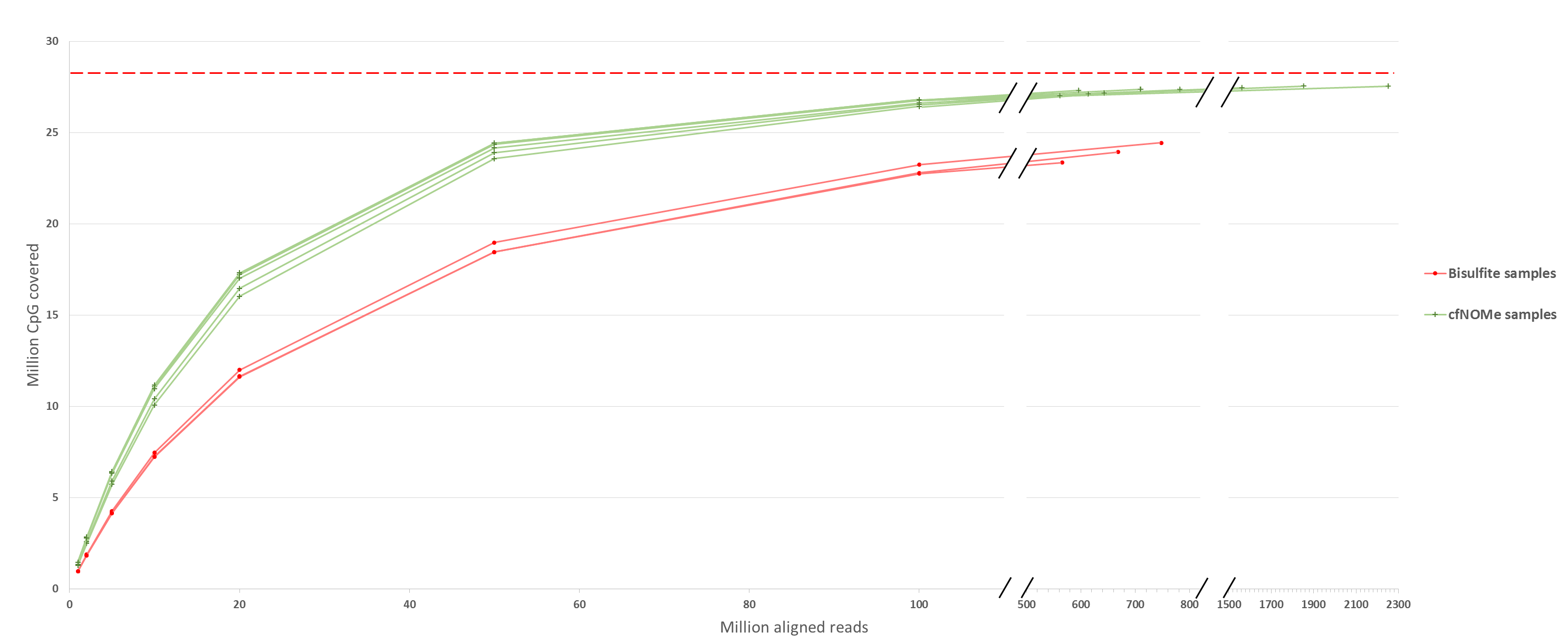


Fig. S12: Number of CpG positions covered at least 1x in cfNOMe vs. Bisulfite sequencing. cfNOMe achieves coverage of more CpG than Bisulfite at the same level of sequencing. Even in very large datasets (>500 million reads), Bisulfite converted libraries leave approximately 4 million CpGs (~14%) uncovered. A total of 28,217,448 CpG genome-wide were studied (represented by dashed red line). Larger sequencing datasets were downsampled to 1×10^6^, 2×10^6^, 5×10^6^, 1×10^7^, 2×10^7^, 5×10^7^ and 1×10^8^ aligned reads. Each CpG was then assessed for the presence of any overlapping read in the downsampled datasets as well as the complete datasets. All datasets are included.


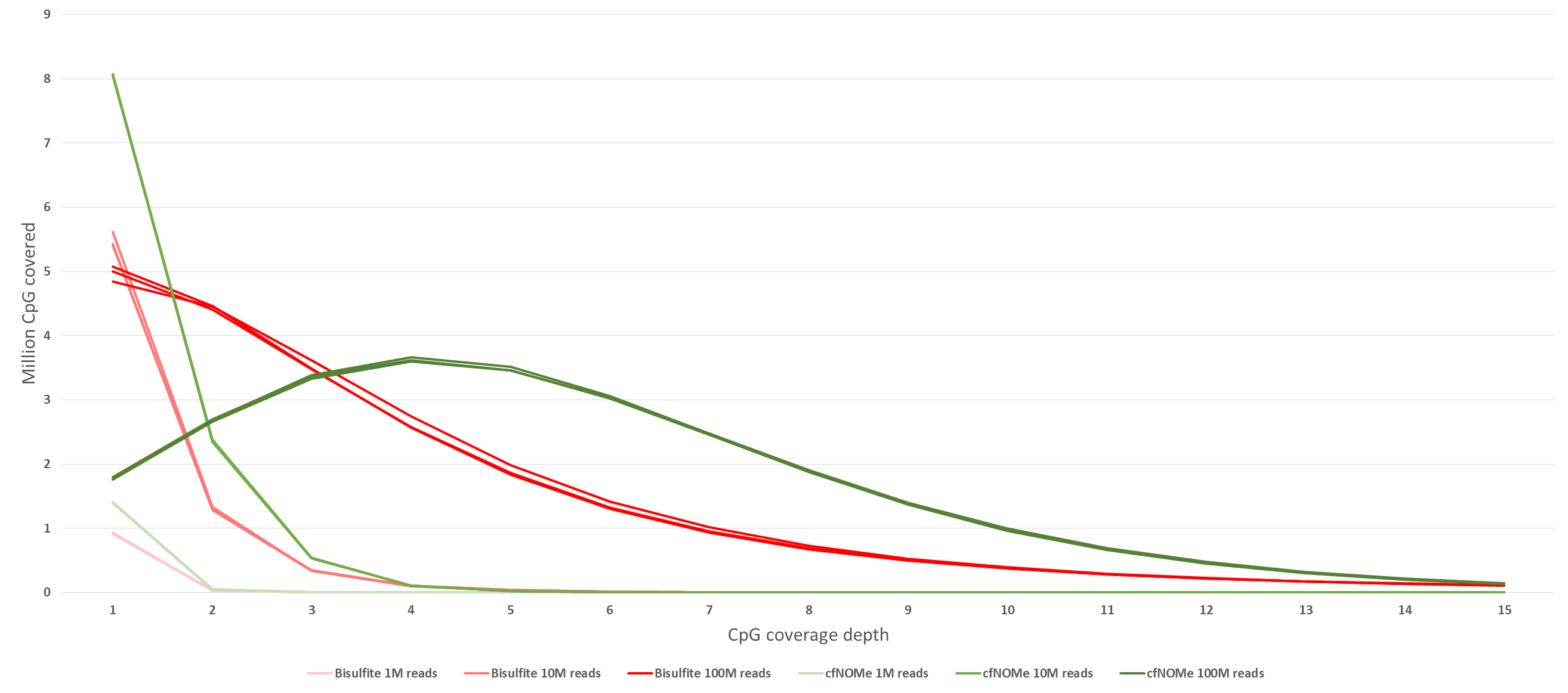


Fig. S13: Coverage depth over analysed CpG positions in cfNOMe vs. Bisulfite sequencing. More CpG positions are more highly covered with increasing dataset size. Larger sequencing datasets were downsampled to 1×10^6^, 1×10^7^ and 1×10^8^ aligned reads for each CpG, the corresponding coverage depth was assessed. Comparison between equally sized replicates of K19, K20 and K21, converted either enzymatically or using bisulfite conversion.


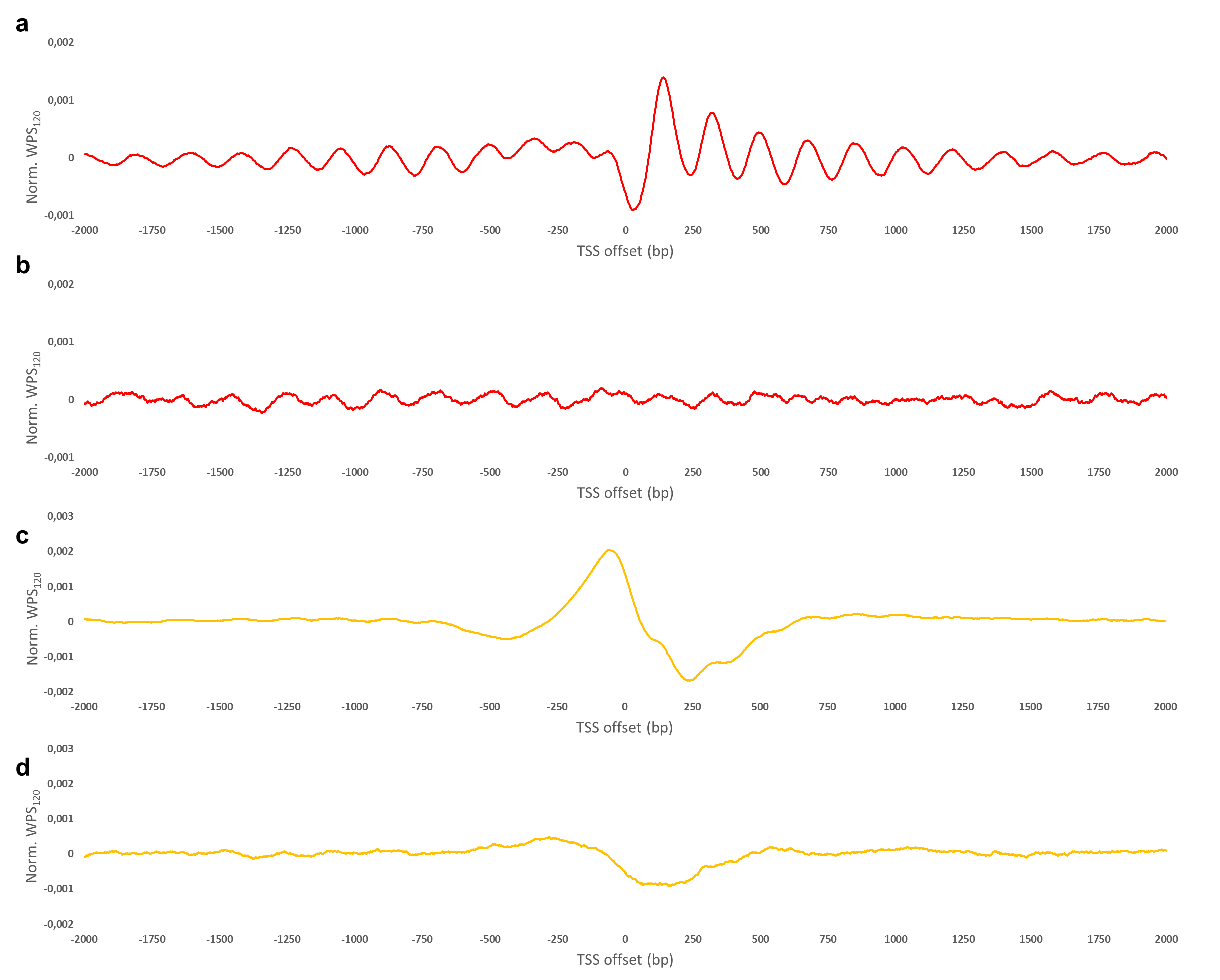


Fig. S14: Comparison of the aggregate normalised WPS_120_ around the transcription start site (TSS) of autosomal genes with any level of expression in whole blood (n=15,368) and autosomal genes with zero expression in whole blood (n=2,447). **a**, Positive whole blood expression genes, blood-derived cfDNA. **b**, Zero whole blood expression genes, blood-derived cfDNA. **c**, Positive whole blood expression genes, urine-derived cfDNA. **d**, Zero whole blood expression genes, urine-derived cfDNA. No signal is visible for genes with zero whole blood expression in blood-derived cfDNA, whereas a weak signal exists for those same genes in urine-derived cfDNA. 1,207 (~49%) of the analysed genes with zero expression in whole blood have positive expression in the kidney. All fragments (1bp-500bp) were studied in the merged datasets of healthy individual K16.


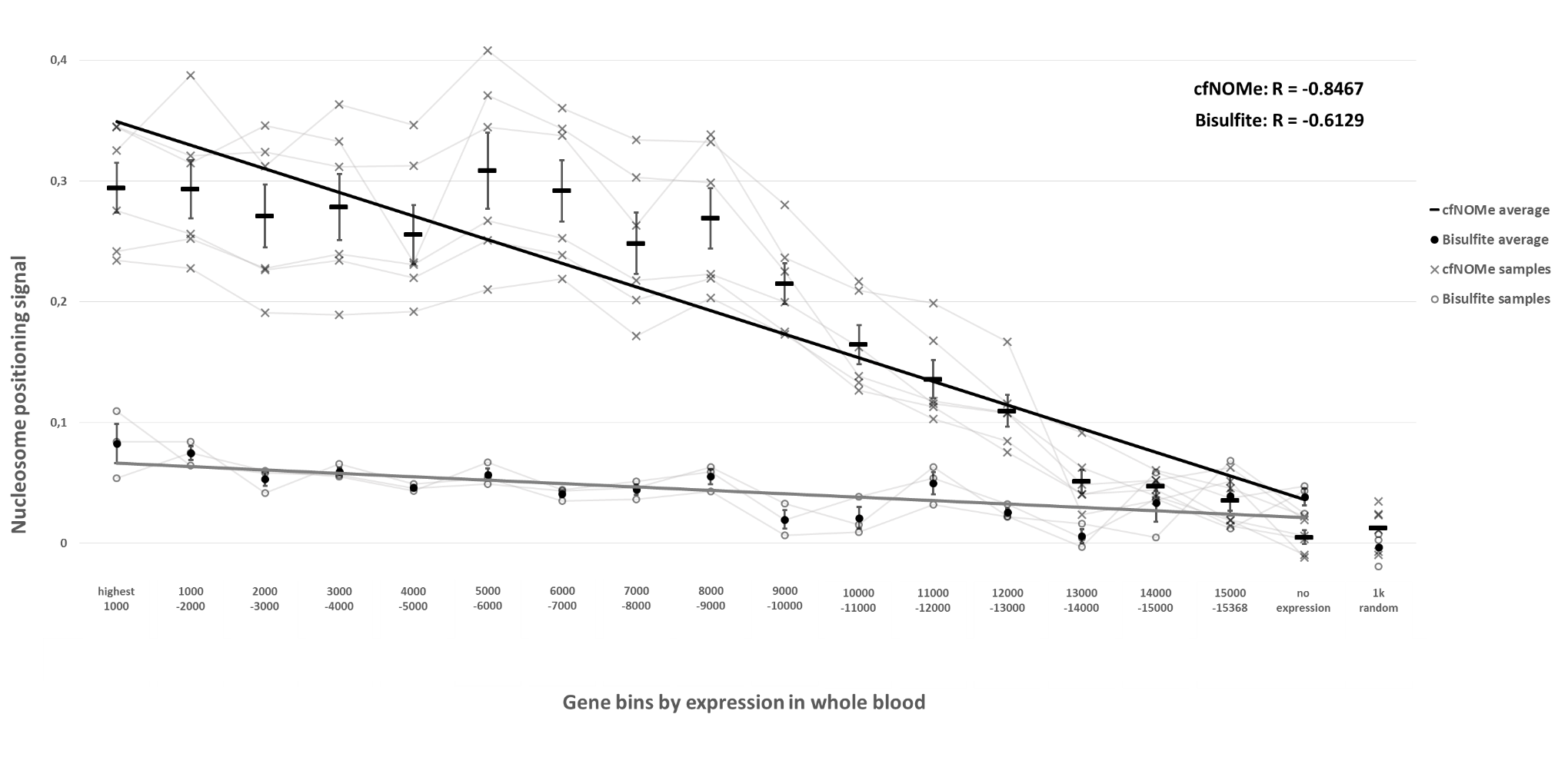

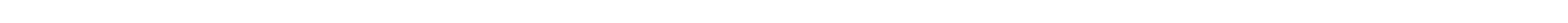


Fig. S15: Correlation between the nucleosome positioning signal (NPS) as measured in blood-derived cfDNA and the level of gene expression in whole blood. For this analysis, the very long cfDNA fragments between 200bp to 500bp were analysed. There is a strong negative correlation apparent in cfNOMe datasets. Bisulfite-converted datasets also show a weaker correlation for this metric at similar sequencing dataset sizes. The NPS for this analysis was calculated as $\sum P_{135..185}-\sum P_{10..60}$ from the respective aggregate WPS_120_ scores. A different offset from the transcription start sites is necessary depending on the chosen WPS window size. Only autosomal genes are studied. The “no expression” category contains 2,447 genes with zero expression in whole blood. The “1k random” category contains 1,000 randomly selected coordinates in the hg19 reference genome. All merged datasets are analysed. Error bars represent SEM.


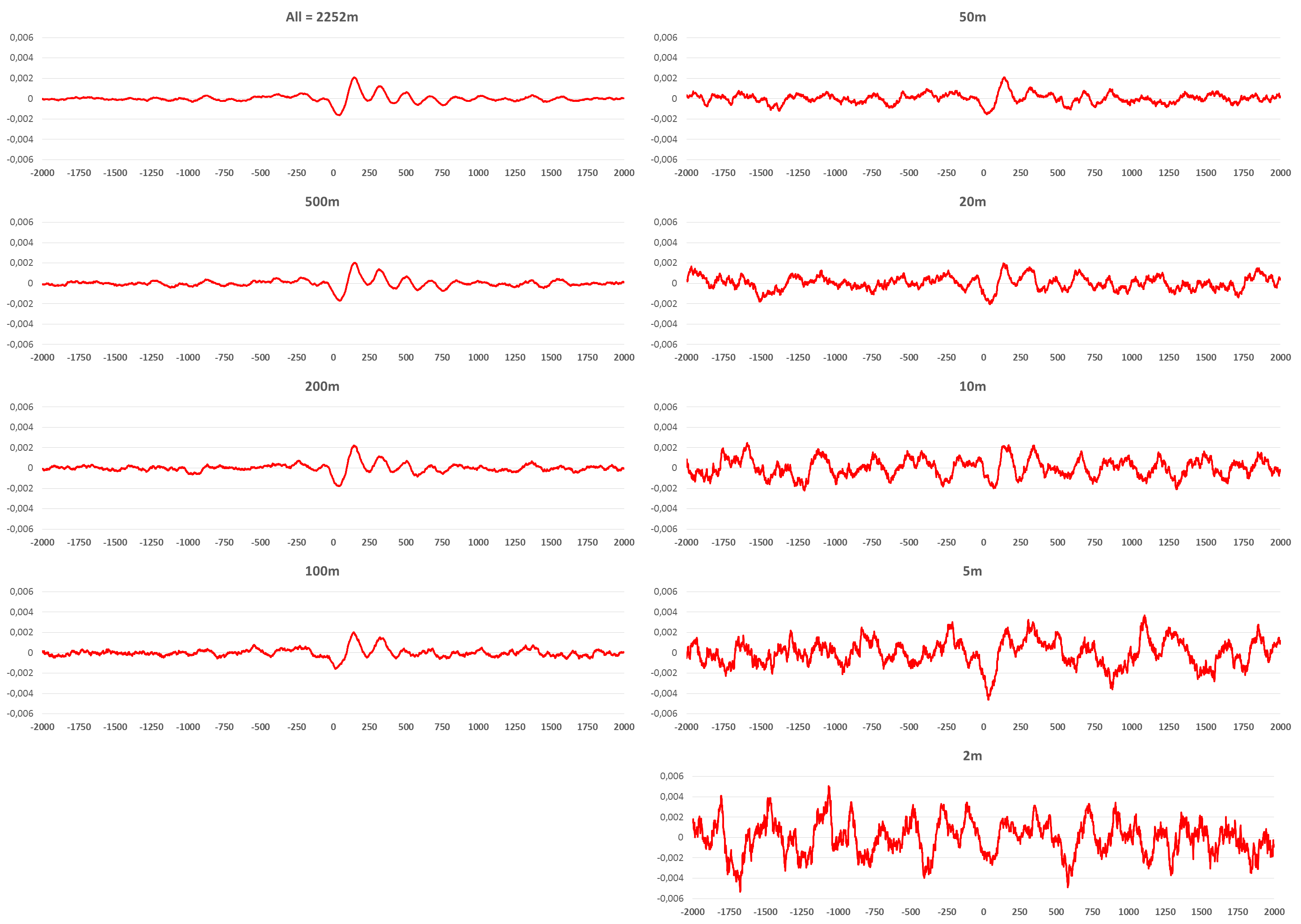


Fig. S16: Downsampling series of windowed protection score (WPS) calculations. The 1000 most highly expressed genes in whole blood were chosen and the aggregate normalised WPS_120_ at their transcription start sites calculated in control sample K16. The complete K16 blood dataset contained ~2.25 billion mapped reads. Further downsampled datasets were generated with sizes between 500 million and 2 million mapped reads. A reliable detection of strong WPS patterns in this context seems to be possible in datasets containing 200 million or more reads. Smaller datasets exhibit increased noise, making downstream analyses more challenging.


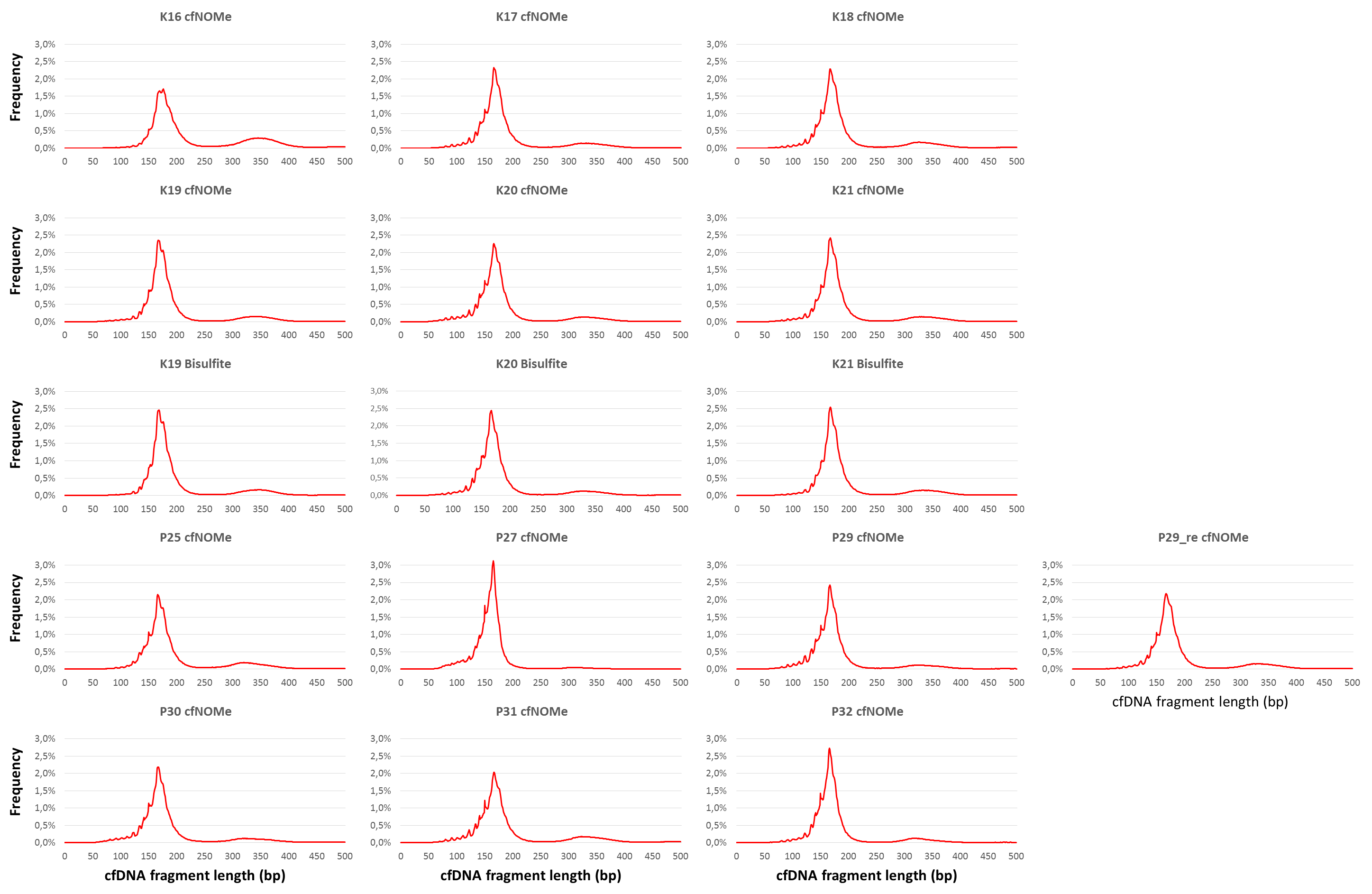


Fig. S17: Blood-derived cfDNA fragment length histograms (n=5,000,000 fragments per library are plotted). The typical fragmentation patterns of cfDNA are maintained in libraries generated with either method. A fragment length peak at ~170bp in blood-derived cfDNA is evident, as well as an additional fragment length periodicity of 10.5bp. The former feature is called the nucleosomal peak and represents the length of DNA wrapped around a single nucleosome including the adjacent linker DNA[2], the latter periodicity represents the rotation of the DNA double helix along its longitudinal axis when wrapped around the nucleosome[3].


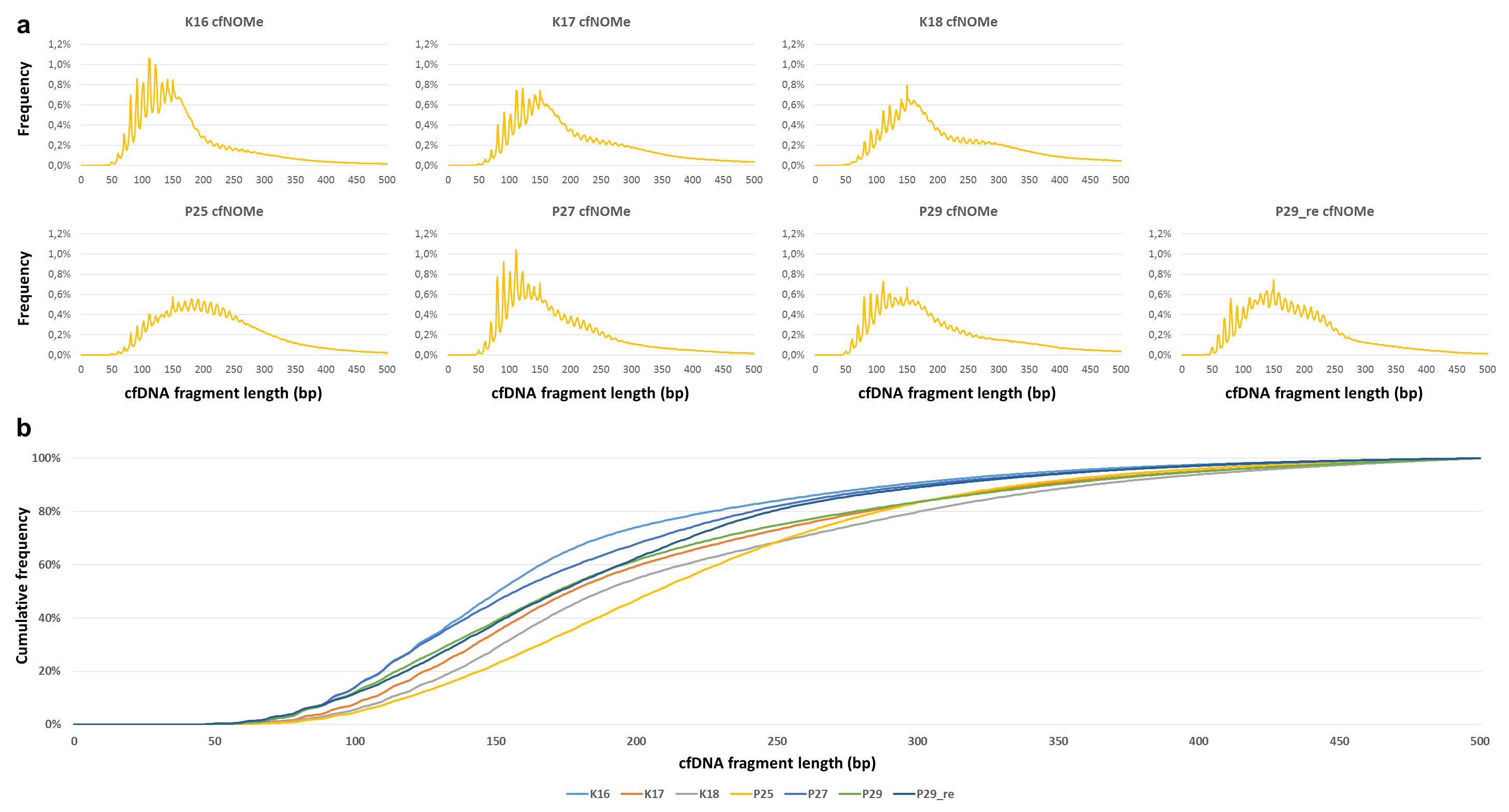
 Fig. S18: **a**, Urine-derived cfDNA fragment length histograms (n=5,000,000 fragments per library are plotted). In the studied urine samples, the nucleosomal peak is shifted left to between 130bp and 160bp, likely due to cfDNA degradation *in vivo*[4]. The 10.5bp periodicity is accentuated. **b**, Cumulative fragment length frequency graph for each sample.


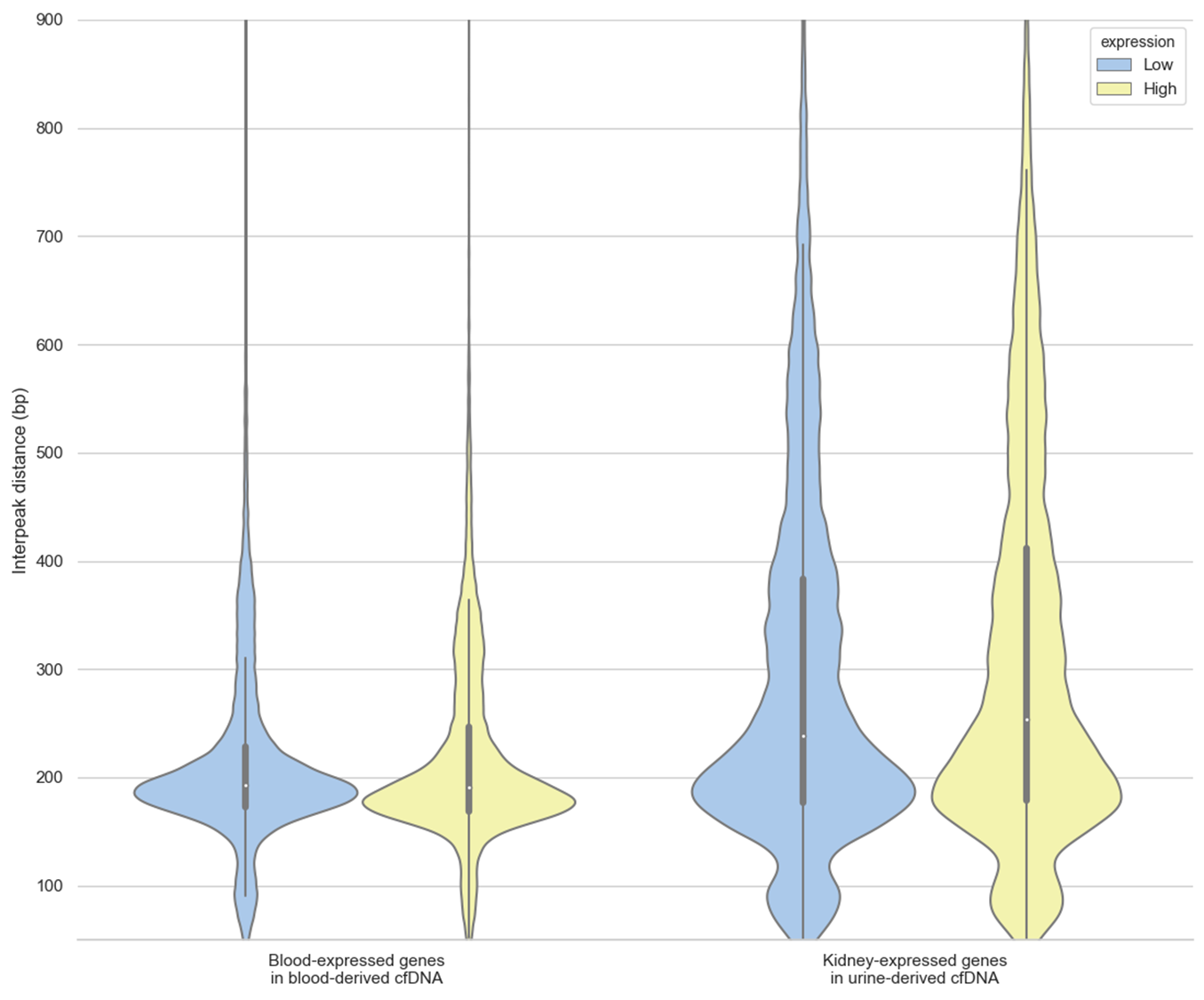
 Fig. S19: Violin plot of the interpeak distances (IPD) in the windowed protection score signal. The IPD within 1kb of the transcription start site of high- and low-expression genes in whole blood (measured in blood-derived cfDNA) and kidney tissue (measured in urine-derived cfDNA) were identified. The graph compares the IPDs around the transcription start site of 2,500 high- vs. 2,500 low-expression genes in the merged dataset of healthy individual K16. A small increase of mean IPD, as well as a larger proportion of long IPDs are visible around the TSS of more highly expressed genes in the urine-derived cfDNA comparison. Thick central line: Q1, Q3; white dot: median; whiskers represent 1.5x interquartile range.


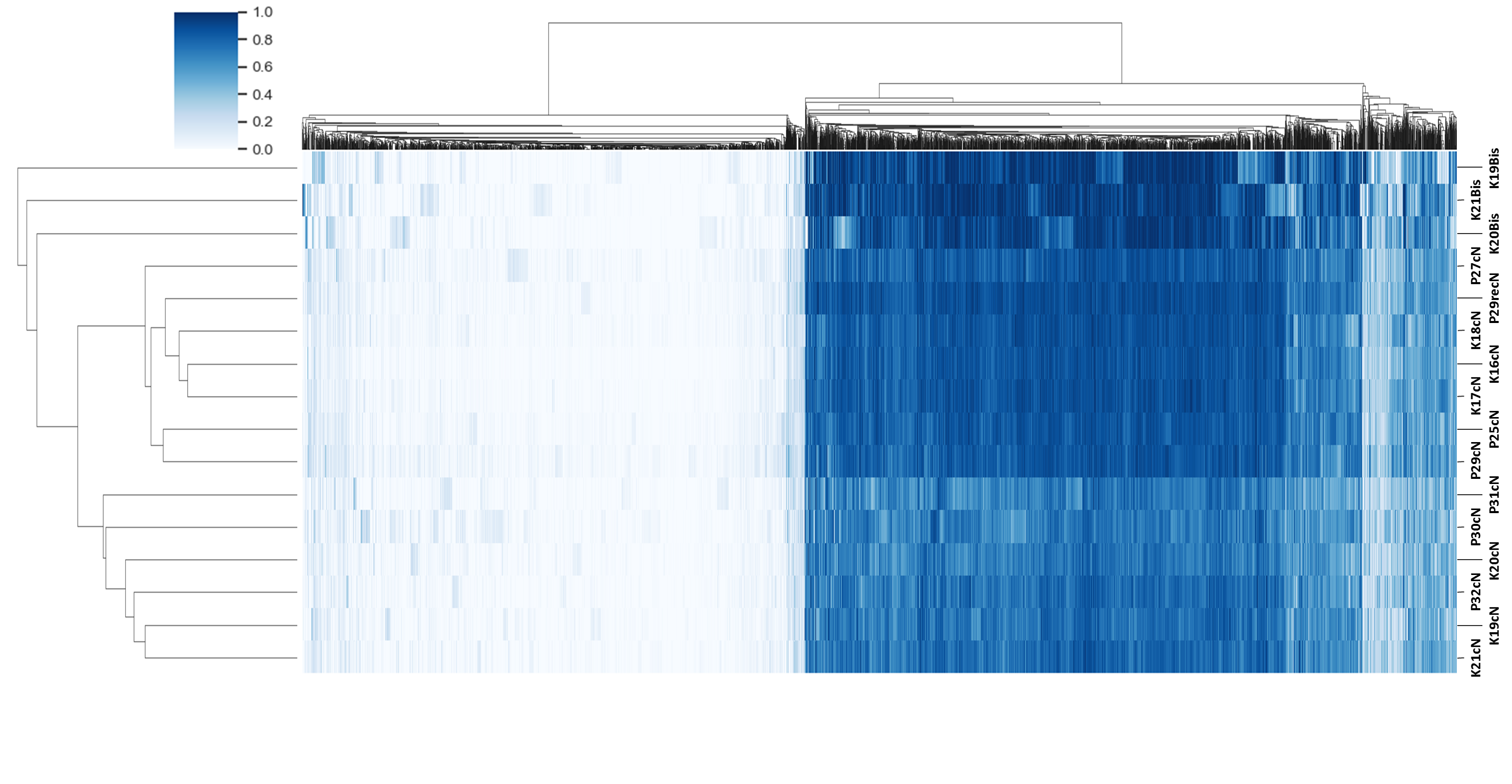


Fig. S20: Methylation profiles at 7890 informative differentially methylated CpGs of all analysed blood-derived cfDNA samples. Bisulfite-converted and enzymatically converted samples, respectively, cluster together. Methylation levels were assessed in merged datasets, where applicable.


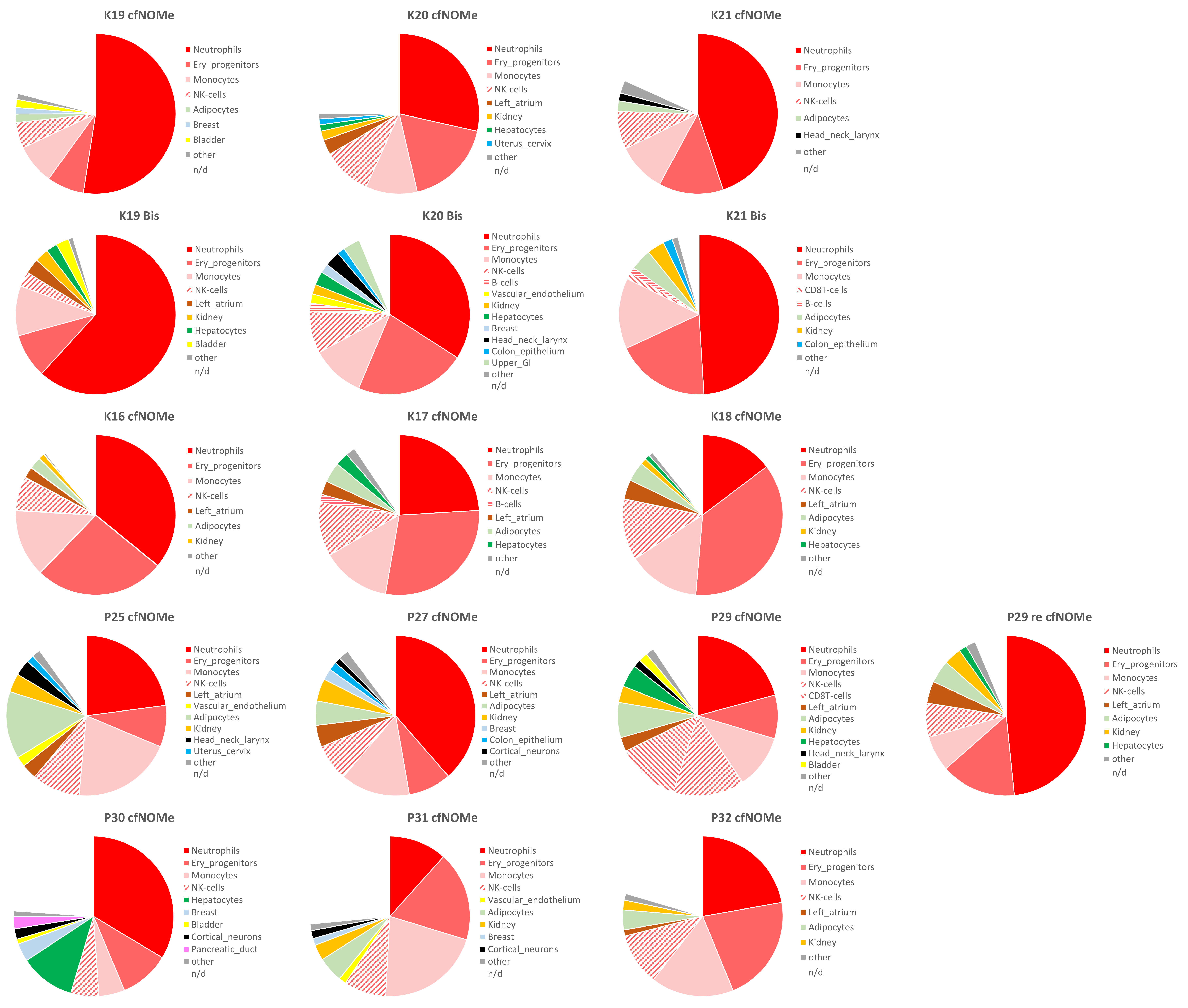


Fig. S21: Methylation deconvolution results for all analysed blood-derived cfDNA samples. Different components of the hematopoietic system make up the majority in all studied datasets. Fractions of ≥1% are shown. Fractions below 1% are summarized under “other”. The displayed fractions represent the best fit under a constraint of the sum of all tissues being less than or equal to 1. Hence, the best fit of tissue proportions may or may not sum up to 1, leaving the remainder without a tissue-of-origin determination (white slices labelled “n/d”). Higher proportions of non-blood cfDNA origins are evident in patients suffering from acute kidney injury or acute kidney graft dysfunction (P25, P27, P29; average±SD non-blood: 23.9%±4.4%) compared to healthy individuals (K16-K21; 9.3%±3.7%) and the post-recovery patient dataset (P29 re). In the patient with acute pancreatitis (P30), cfDNA of pancreatic origin is recognized.


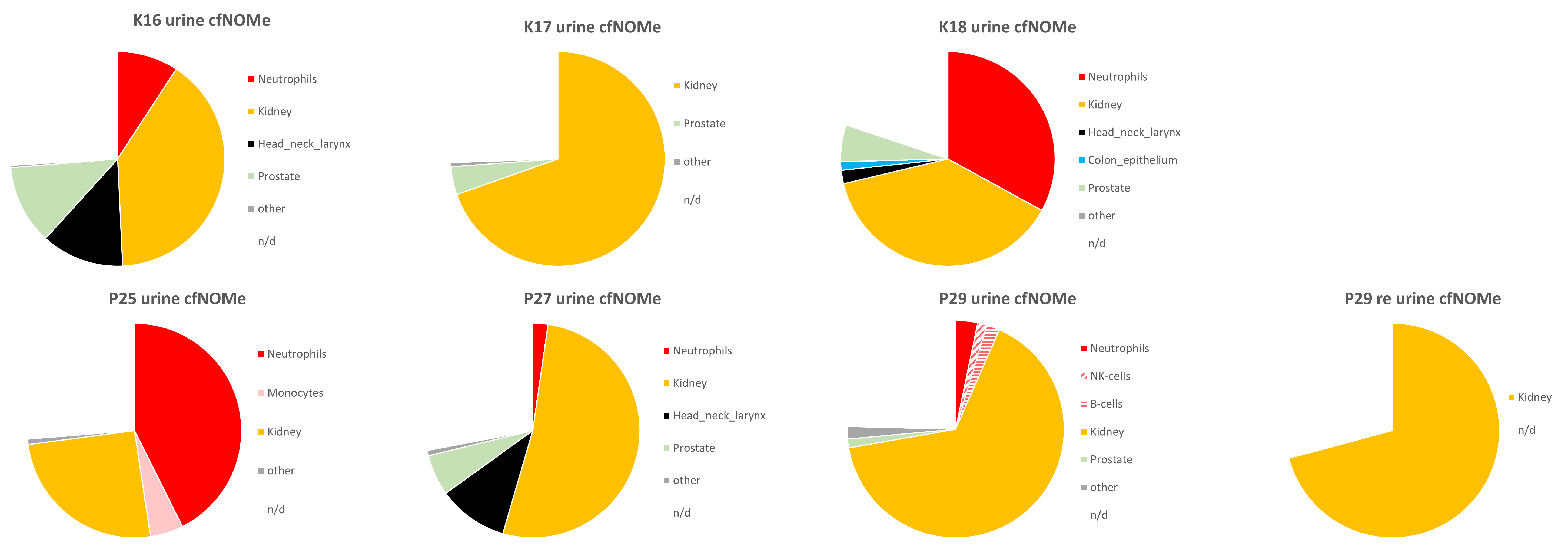


Fig. S22: Methylation deconvolution results for all analysed urine-derived cfDNA samples. cfDNA fractions originating from immune cells and kidney cells make up the majority. The proportion of renal cfDNA varies widely. Smaller proportions of biologically implausible fractions like “Head_neck_larynx” and “Prostate” in female individuals are likely artefactual, caused by these tissues’ reference methylation profiles’ proximity to “Bladder” and “Kidney” (Fig. S9).


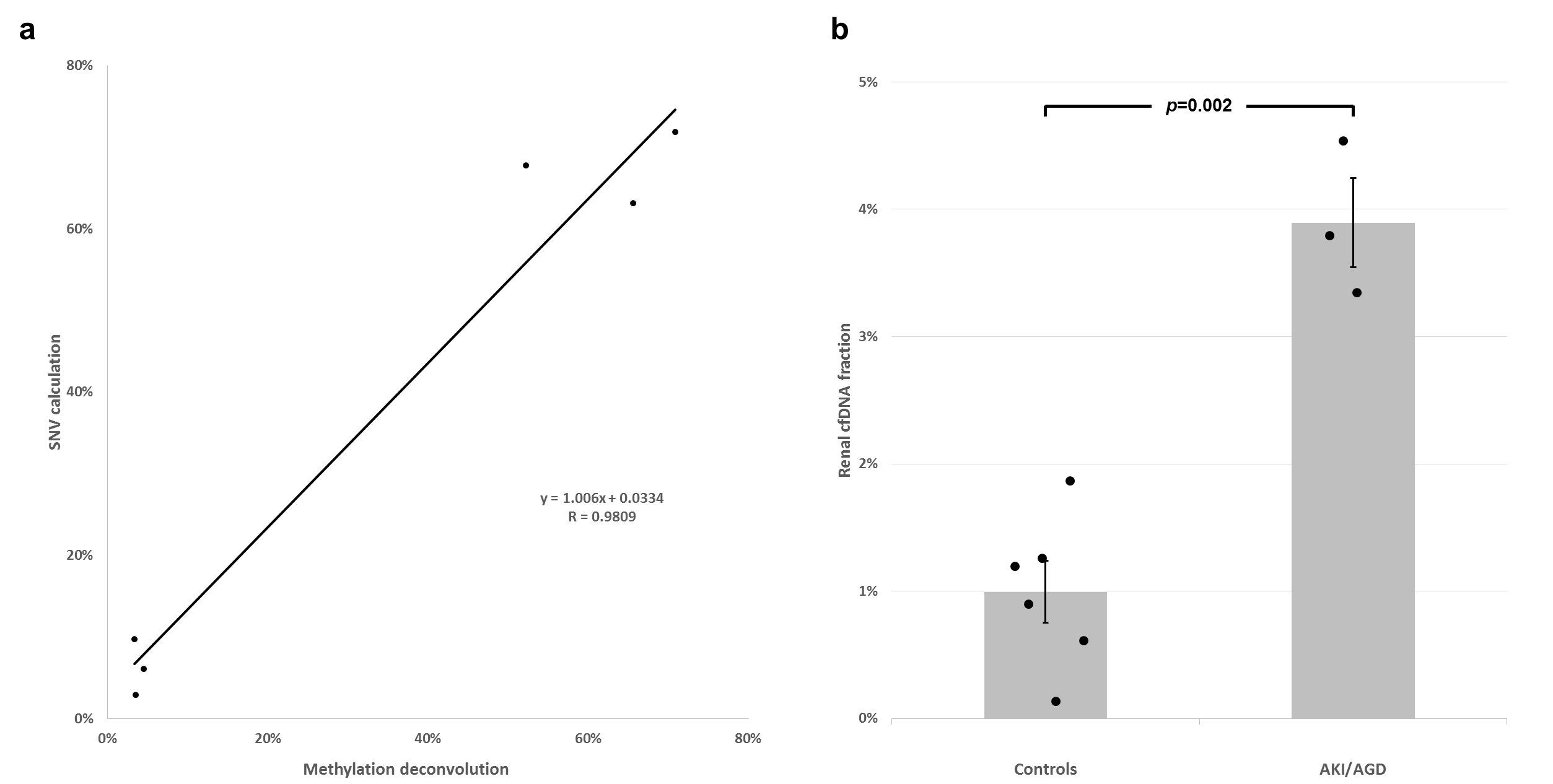


Fig. S23: **a**, Scatterplot of renal cfDNA fractions calculated from methylation deconvolution vs. calculated from donor SNPs (measurements only possible in patients with kidney transplant: P27, P29 and P29 resampling in both blood and urine, n=6 measurement pairs). For P27, a total of 1,326 SNP were used, for P29 a total of 776 SNPs. **b**, Deconvolution-calculated renal cfDNA fraction in blood in healthy controls K16-K21 (n=6) vs. patients P25, P27 and P29 (n=3). Error bars represent SEM. AKI: acute kidney injury, AGD: acute graft dysfunction.


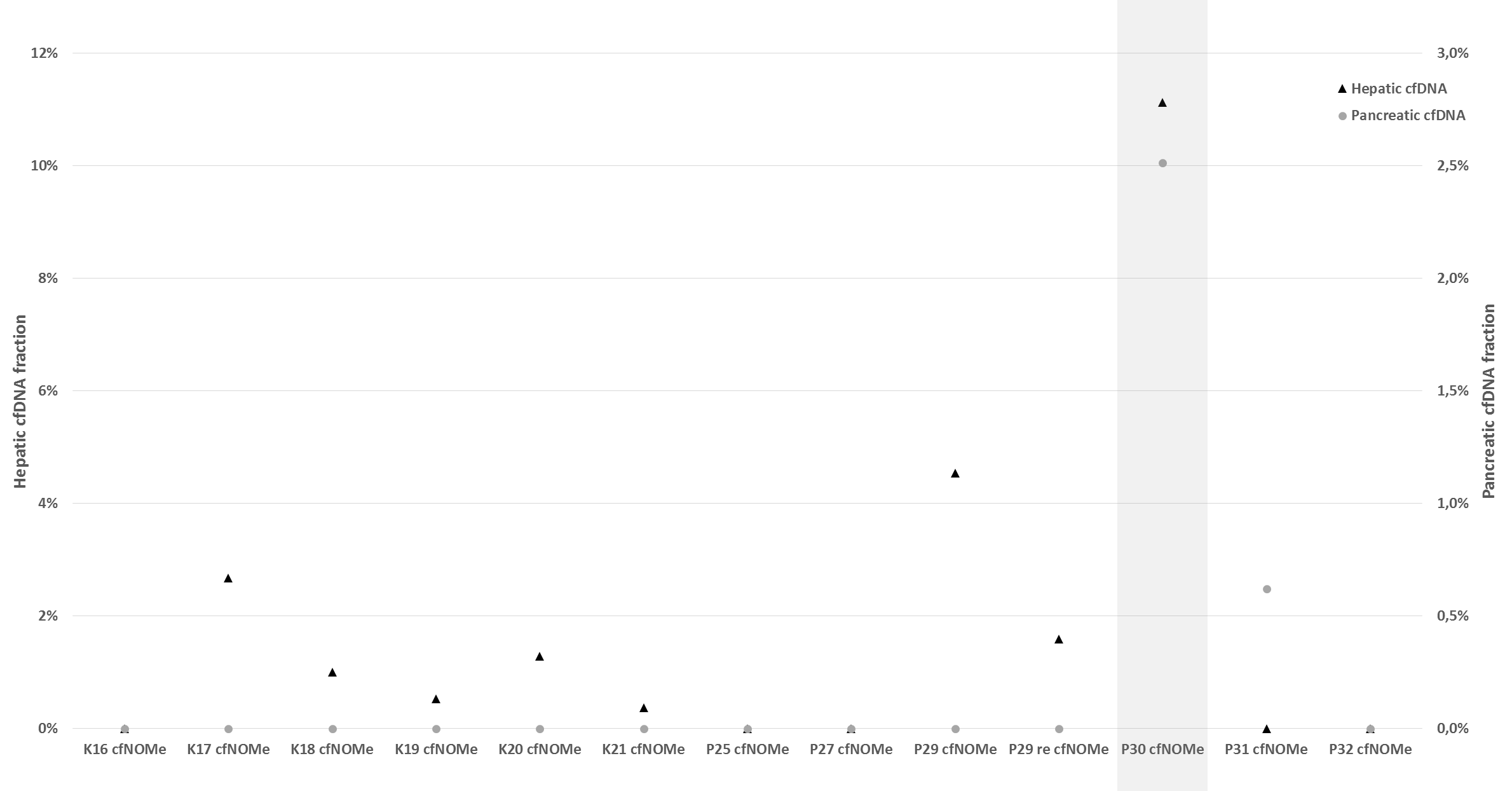


Fig. S24: Deconvolution-calculated cfDNA fractions with liver and pancreas tissue-of-origin. The highest levels of hepatic (~11%) and pancreatic (~2.5%) cfDNA were observed in patient P30 with acute necrotising pancreatitis and elevated liver enzymes (Table S8).


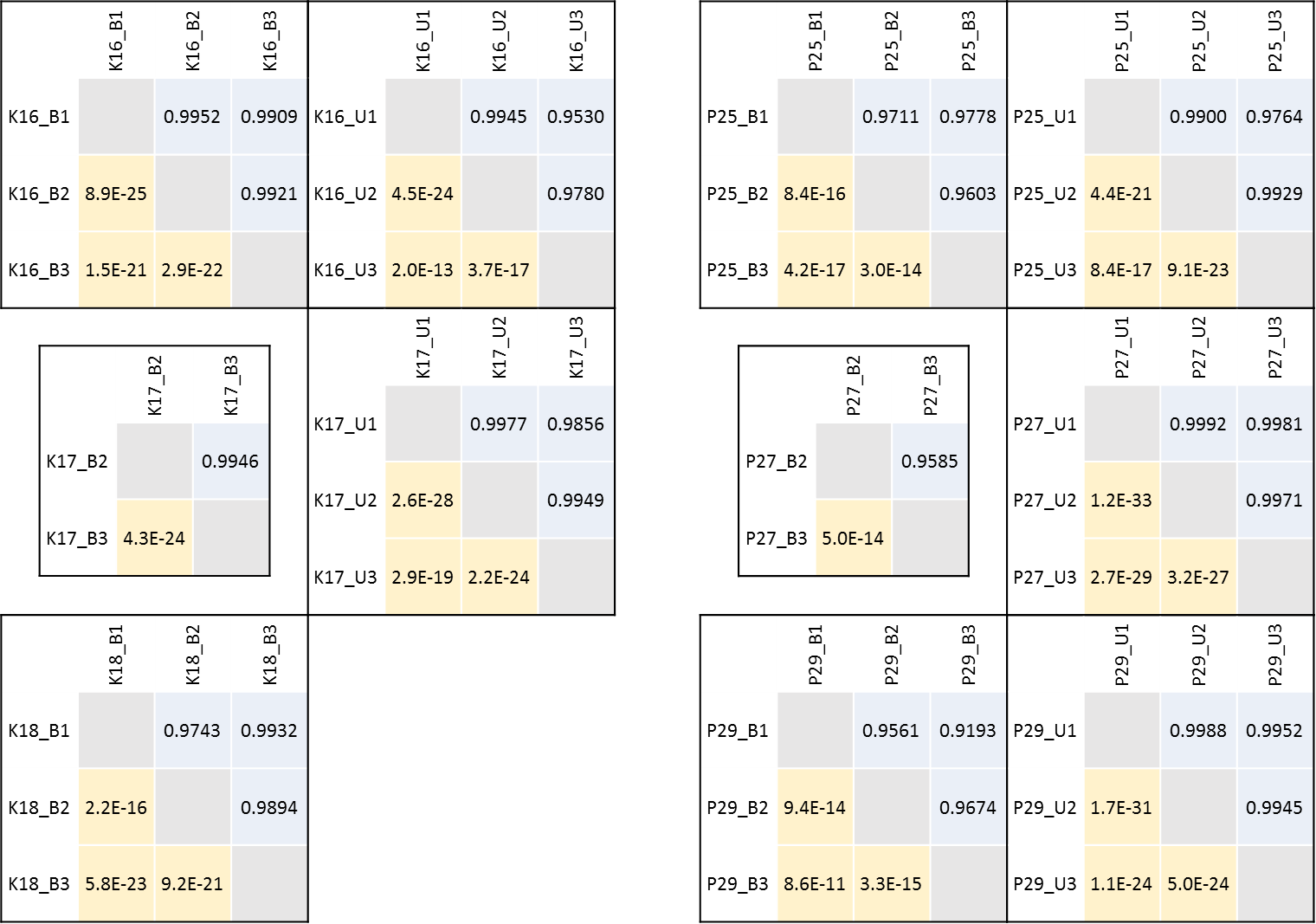


Fig. S25: Deconvolution results: pair-wise comparison of all replicates for each sample from the discovery cohort, where possible. Left: healthy control samples. Right: acute kidney injury/graft dysfunction (AKI/AGD) patient samples. For the control sample K17 and patient sample P27, only two replicates of blood-derived cfDNA were sequenced. The urine-derived cfDNA of K18 was sequenced from a single library. The samples of healthy control K17 were collected on subsequent days at 8:00am, noon, and 9:30pm. K16, K17, K18: healthy controls. P25, P27, P29: AKI/AGD patients. Blood cfDNA samples are marked “B”, urine cfDNA samples are marked “U”. Light-blue shading: Pearson’s R correlation coefficient, yellow shading: associated p-values.

Table S1: Library preparation steps and DNA amounts in urine samples.

| Tissue | Sample ID | DNA concentration after extraction (ng/µl) | Volume (µl) | Total DNA pre-conversion (ng) | Conversion kit | PCR cycles | Library concentration (ng/µl) | Total DNA (ng) |
| --- | --- | --- | --- | --- | --- | --- | --- | --- |
| **Urine** | K16_U1 | 1.28 | 40 | 51.2 | NEB EM-Seq | 6 | 4.19 | 83.8 |
|  | K16_U2 | 1.34 | 40 | 53.6 | NEB EM-Seq | 6 | 2.54 | 50.8 |
|  | K16_U3 | 1.41 | 40 | 56.4 | NEB EM-Seq | 6 | 6.34 | 126.8 |
|  | K17_U1 | 3.28 | 40 | 131.2 | NEB EM-Seq | 5 | 10.7 | 214 |
|  | K17_U2 | 1.28 | 40 | 51.2 | NEB EM-Seq | 6 | 4.91 | 98.2 |
|  | K17_U3 | 2.38 | 40 | 95.2 | NEB EM-Seq | 5 | 4.48 | 89.6 |
|  | K18_U1 | 6.78 | 40 | 271.2 | NEB EM-Seq | 4 | 1.53 | 30.6 |
|  | P25_U1 | 7.34 | 40 | 293.6 | NEB EM-Seq | 4 | 7.34 | 146.8 |
|  | P25_U2 | 7.58 | 40 | 303.2 | NEB EM-Seq | 4 | 7.58 | 151.6 |
|  | P25_U3 | 7.4 | 40 | 296 | NEB EM-Seq | 4 | 8.4 | 168 |
|  | P27_U1 | 1.39 | 40 | 55.6 | NEB EM-Seq | 6 | 3.29 | 65.8 |
|  | P27_U2 | 1.33 | 40 | 53.2 | NEB EM-Seq | 6 | 3.34 | 66.8 |
|  | P27_U3 | 1.41 | 40 | 56.4 | NEB EM-Seq | 6 | 3.08 | 61.6 |
|  | P29_U1 | 1.06 | 40 | 42.4 | NEB EM-Seq | 6 | 1.29 | 25.8 |
|  | P29_U2 | 1.07 | 40 | 42.8 | NEB EM-Seq | 6 | 1.14 | 22.8 |
|  | P29_U3 | 1.14 | 40 | 45.6 | NEB EM-Seq | 6 | 1.3 | 26 |

Table S2: Library preparation steps and DNA amounts in blood samples, discovery cohort.

| Tissue | Sample ID | DNA concentration after extraction (ng/µl) | Volume (µl) | Total DNA pre-conversion (ng) | Conversion kit | PCR cycles | Library concentration (ng/µl) | Total DNA (ng) |
| --- | --- | --- | --- | --- | --- | --- | --- | --- |
| **Blood** | K16_B1 | 0.884 | 40 | 35.36 | NEB EM-Seq | 7 | 15.1 | 302 |
|  | K16_B2 | 0.928 | 40 | 37.12 | NEB EM-Seq | 7 | 12.8 | 256 |
|  | K16_B3 | 0.844 | 40 | 33.76 | NEB EM-Seq | 7 | 9.71 | 194.2 |
|  | K17_B2 | 0.932 | 40 | 37.28 | NEB EM-Seq | 7 | 3.96 | 79.2 |
|  | K17_B3 | 0.714 | 40 | 28.56 | NEB EM-Seq | 7 | 2.65 | 53 |
|  | K18_B1 | 0.752 | 40 | 30.08 | NEB EM-Seq | 7 | 2.8 | 56 |
|  | K18_B2 | 0.974 | 40 | 38.96 | NEB EM-Seq | 7 | 5.2 | 104 |
|  | K18_B3 | 0.92 | 40 | 36.8 | NEB EM-Seq | 7 | 8.97 | 179.4 |
|  | P25_B1 | 1.29 | 40 | 51.6 | NEB EM-Seq | 6 | 4.77 | 95.4 |
|  | P25_B2 | 1.4 | 40 | 56 | NEB EM-Seq | 6 | 4.93 | 98.6 |
|  | P25_B3 | 1.43 | 40 | 57.2 | NEB EM-Seq | 6 | 3.55 | 71 |
|  | P27_B2 | 24.2 | 40 | 968* | NEB EM-Seq | 4 | 6.56 | 131.2 |
|  | P27_B3 | 26.4 | 40 | 1056* | NEB EM-Seq | 4 | 14.4 | 288 |
|  | P29_B1 | 1.61 | 40 | 64.4 | NEB EM-Seq | 6 | 7.25 | 145 |
|  | P29_B2 | 1.51 | 40 | 60.4 | NEB EM-Seq | 6 | 7.76 | 155.2 |
|  | P29_B3 | 1.72 | 40 | 68.8 | NEB EM-Seq | 6 | 8.57 | 171.4 |

*: 200ng DNA were used for the subsequent conversion.

Table S3: Library preparation steps and DNA amounts in blood samples, replication cohort.

| Sample ID | DNA concentration after extraction (ng/µl) | Volume (µl) | Total DNA pre-conversion (ng) | methylated pUC19 DNA (µl) | unmethylated lambda DNA (µl) | Conversion Kit | PCR cycles | Library concentration (ng/µl) | Total DNA (ng) |
| --- | --- | --- | --- | --- | --- | --- | --- | --- | --- |
| K19 | 1.18 | 80 | 94.4 | 4.8 | 4.8 | Epitect Bisulfite | 6 | 2.50 | 50.0 |
|  |  |  |  |  |  | NEB EM-Seq | 6 | 2.58 | 51.6 |
| K20 | 0.235 | 80 | 18.8 | 1.0 | 1.0 | Epitect Bisulfite | 8 | 1.99 | 39.8 |
|  |  |  |  |  |  | NEB EM-Seq | 8 | 1.57 | 31.4 |
| K21 | 0.962 | 80 | 77.0 | 3.8 | 3.8 | Epitect Bisulfite | 6 | 2.00 | 40.0 |
|  |  |  |  |  |  | NEB EM-Seq | 6 | 1.87 | 37.4 |
| P30 | 2.55 | 40 | 102.0 | 5.1 | 5.1 | NEB EM-Seq | 5 | 3.27 | 65.4 |
| P31 | 0.259 | 40 | 10.4 | 0.5 | 0.5 | NEB EM-Seq | 8 | 0.87 | 17.5 |
| P32 | 1.13 | 40 | 45.2 | 2.3 | 2.3 | NEB EM-Seq | 6 | 2.99 | 59.8 |

Table S4: Reaction composition using PyroMark PCR Master Mix. Reagents, concentrations and volumes per reaction required for the PyroMark PCR are shown.

| **Reagent** | **Input concentration** | **Volume per reaction** |
| --- | --- | --- |
| **DNA** | 6 ng/µl | 5 µl |
| **Controls (10ng/µl)** | 2 ng/µl | 3 µl |
| **PyroMark PCR Master mix (2x)** | 1x | 7.5 µl |
| **Coral Load concentrate (10x)** | 1x | 1.5 µl |
| **Primer forward** | 0.2 µM | 0.3 µl |
| **Primer reverse** | 0.2 µM | 0.3 µl |
| **ddH_2_O** |  | 0.4 µl |

Table S5: PCR protocol for PyroMark PCR reaction. All PCRs were performed with 45 cycles, annealing temperatures were adjusted according to the primers used (see Table S6).

| **Step** | **Duration** | **Temperature** |
| --- | --- | --- |
| **Initial PCR activation step** | 15 min | 95°C |
| **Denaturation** | 30 sec | 94°C |
| **Annealing** | 30 sec | variable |
| **Extension** | 30 sec | 72°C |
| **Number of cycles** | 45x |  |
| **Final extension** | 10 min | 72°C |

Table S6: Primer sequences and reaction conditions for loci of interest in *SLC34A1*, *UMOD* and *CTXN3*. Red ‘R’s mark positions of analysed cytosines. The genomic positions analysed in *SLC34A1* are (hg19) chr5:176812116 (locus 1), chr5:176812123 (locus 2) and chr5:176812139 (locus 3), in *UMOD* chr16:20364262 (locus 1) and chr16:20364284 (locus 2) and in *CTXN3* chr5:126988758 (locus 1). A sequenced ‘A’ in those positions refers to an unmethylated cytosine in the template (which is read as ‘T’ by the polymerase), a sequenced ‘G’ to a methylated cytosine in the template (which is read as ‘C’ by the polymerase; see Fig. S1).

| **SLC34A1** | |
| --- | --- |
| **Primer F** | biotin-GGGAAAGGAATTTATTGTGA |
| **Primer R** | CAAACACCTACCTTCTCATAA |
| **Pyrosequencing primer** | AAAATCCCCTAAACC |
| **Annealing Temp** | 52°C |
| **Sequence 1** | **R**AATCCC**R**AATTCCTAAATTCCCCTCACAATAA |
| **Sequence 2** | CACAC**R**AAAATCCCCTAAACC |
| **UMOD** | |
| **Primer F** | TTGGGGGGTTATATTTGTGT |
| **Primer R** | biotin-AACCCAACACCTTAACACTACTT |
| **Pyrosequencing primer** | GGGGGGTTATATTTGTG |
| **Annealing Temp** | 57°C |
| **Sequence** | TT**R**GGAAATATAAGGTTATTGTTT**R**GTA |
| **CTXN3** | |
| **Primer F** | TGTGGATATTGGGGTGTAAAGG |
| **Primer R** | biotin-TCCTCATCCCTTACCCATACTTC |
| **Pyrosequencing primer** | TGGGTTTAAAAAGATGAAT |
| **Annealing Temp** | 60°C |
| **Sequence** | **R**GAGATGTAGTGTGTAGGAAGTATGG |

Table S7: Sequencing and associated clinical data for each sample in this study, discovery cohort.

| **Sample name** | **Clinical data** | **Sample description** | **Sample ID** | **Barcode sequence** | **PF Clusters** | **Yield (Gbases)** | **PE reads for alignment** | **Uniquely mapped reads** | **Estimated hg19 coverage** | **% >= Q30 bases** | **Mean Quality**  **Score** |
| --- | --- | --- | --- | --- | --- | --- | --- | --- | --- | --- | --- |
| K16 | Healthy   Adult female | K16, blood cfDNA, EMseq, triplicate | 91958 | TGACCA | 467,401,072 | 141.2 | 934,802,144 |  |  | 90.10 | 35.27 |
|  |  |  | 91959 | ACAGTG | 421,277,761 | 127.2 | 842,555,522 |  |  | 90.34 | 35.32 |
|  |  |  | 91960 | GCCAAT | 534,113,263 | 161.3 | 1,068,226,526 |  |  | 90.22 | 35.29 |
|  |  | ∑ |  |  |  | **429.7** |  | 2,252,119,902 | 109.0 |  |  |
|  |  | K16, urine cfDNA, EMseq, triplicate | 94314 | AGTCAA | 429,299,057 | 129.7 | 858,598,114 |  |  | 88.88 | 34.98 |
|  |  |  | 94315 | AGTTCC | 451,257,565 | 136.3 | 902,515,130 |  |  | 89.19 | 35.05 |
|  |  |  | 94316 | ATGTCA | 379,359,910 | 114.6 | 758,719,820 |  |  | 88.81 | 34.96 |
|  |  | ∑ |  |  |  | **380.6** |  | 1,968,803,556 | 95.3 |  |  |
| K17 | Healthy   Adolescent male | K17, blood cfDNA, EMseq,  different time points | 94321 | GTTTCG | 581,574,772 | 175.6 | 1,163,149,544 |  |  | 90.04 | 35.24 |
|  |  |  | 94322 | CGTACG | 563,273,181 | 170.1 | 1,126,546,362 |  |  | 90.39 | 35.31 |
|  |  | ∑ |  |  |  | **345.7** |  | 1,853,252,068 | 89.7 |  |  |
|  |  | K17, urine cfDNA, EMseq,  different time points | 94317 | CCGTCC | 456,826,086 | 138.0 | 913,652,172 |  |  | 89.82 | 35.19 |
|  |  |  | 94318 | GTCCGC | 479,536,832 | 144.8 | 959,073,664 |  |  | 89.42 | 35.09 |
|  |  |  | 94319 | GTGAAA | 398,090,769 | 120.2 | 796,181,538 |  |  | 89.74 | 35.17 |
|  |  | ∑ |  |  |  | **403.0** |  | 1,944,965,694 | 94.1 |  |  |
| K18 | Healthy   Adult female | K18, blood cfDNA, EMseq, triplicate | 98994 | ATCACG | 245,698,731 | 74.2 | 491,397,462 |  |  | 90.64 | 35.35 |
|  |  |  | 98995 | CGATGT | 344,840,712 | 104.1 | 689,681,424 |  |  | 91.12 | 35.45 |
|  |  |  | 98996 | TTAGGC | 361,797,203 | 109.3 | 723,594,406 |  |  | 90.01 | 35.24 |
|  |  | ∑ |  |  |  | **287.6** |  | 1,562,114,416 | 75.6 |  |  |
|  |  | K18, urine cfDNA, EMseq, single library | 98914 | ATTCCT | 1,030,635,865 | **311.3** | 2,061,271,730 | 1,254,977,530 | 60.4 | 88.63 | 34.94 |
| P25 | Acute kidney injury, rapid-progressive glomerulonephritis, S-Creatinin 6.83mg/dl (Ref <1.1mg/dl), subsequently developed end-stage renal disease  Adolescent male | P25, blood cfDNA, EMseq, triplicate | 94562 | TAGCTT | 603,170,052 | 182.2 | 1,206,340,104 |  |  | 92.05 | 35.61 |
|  |  |  | 94563 | GGCTAC | 506,214,715 | 152.9 | 1,012,429,430 |  |  | 91.71 | 35.55 |
|  |  |  | 94564 | CTTGTA | 511,781,086 | 154.6 | 1,023,562,172 |  |  | 91.77 | 35.56 |
|  |  | ∑ |  |  |  | **489.7** |  | 2,638,721,524 | 127.7 |  |  |
|  |  | P25, urine cfDNA, EMseq, triplicate | 94559 | CAGATC | 293,117,211 | 88.5 | 586,234,422 |  |  | 91.18 | 35.44 |
|  |  |  | 94560 | ACTTGA | 370,185,886 | 111.8 | 740,371,772 |  |  | 91.20 | 35.4 |
|  |  |  | 94561 | GATCAG | 338,355,011 | 102.2 | 676,710,022 |  |  | 91.57 | 35.51 |
|  |  | ∑ |  |  |  | **302.5** |  | 1,430,049,474 | 69.2 |  |  |
| P27 | Acute kidney graft dysfunction, Stage II humoral graft rejection BK-virus nephropathy S-Creatinin 1.4mg/dl → 3.1mg/dl  Adult female | P27, blood cfDNA, EMseq, duplicate | 98904 | AGTTCC | 286,547,663 | 86.5 | 573,095,326 |  |  | 90.78 | 35.35 |
|  |  |  | 98905 | ATGTCA | 351,744,002 | 106.2 | 703,488,004 |  |  | 90.50 | 35.28 |
|  |  | ∑ |  |  |  | **192.8** |  | 1,029,285,312 | 49.8 |  |  |
|  |  | P27, urine cfDNA, EMseq, triplicate | 98900 | TGACCA | 339,111,600 | 102.4 | 678,223,200 |  |  | 89.42 | 35.04 |
|  |  |  | 98901 | ACAGTG | 273,098,101 | 82.5 | 546,196,202 |  |  | 90.04 | 35.18 |
|  |  |  | 98902 | GCCAAT | 302,327,371 | 91.3 | 604,654,742 |  |  | 90.01 | 35.18 |
|  |  | ∑ |  |  |  | **276.2** |  | 1,322,457,494 | 64.0 |  |  |
|  | n/a; donor | P27, donor WGS | 99228 | TAAGTGGT+CTTAAGCC | 320,464,601 | **96.8** | 640,929,202 | 551,138,646 | 26.7 | 92.80 | 35.73 |
| P29 | Acute kidney graft dysfunction, Humoral graft rejection S-Creatinin 4.74mg/dl  Adult male | P29, blood cfDNA, EMseq, triplicate | 98909 | GTGGCC | 372,698,363 | 112.6 | 745,396,726 |  |  | 91.80 | 35.58 |
|  |  |  | 98910 | GTTTCG | 265,555,129 | 80.2 | 531,110,260 |  |  | 91.02 | 35.47 |
|  |  |  | 98911 | CGTACG | 305,441,335 | 92.2 | 610,882,670 |  |  | 91.47 | 35.51 |
|  |  | ∑ |  |  |  | **285.0** |  | 1,568,352,168 | 75.9 |  |  |
|  |  | P29, urine cfDNA, EMseq, triplicate | 98906 | CCGTCC | 344,403,804 | 104.0 | 688,807,608 |  |  | 90.98 | 35.38 |
|  |  |  | 98907 | GTCCGC | 341,104,096 | 103.0 | 682,208,192 |  |  | 91.08 | 35.40 |
|  |  |  | 98908 | GTGAAA | 301,911,334 | 91.2 | 603,822,668 |  |  | 90.62 | 35.30 |
|  |  | ∑ |  |  |  | **298.2** |  | 1,503,182,978 | 72.7 |  |  |
|  | Resampling after improvement of kidney function  S-Creatinin 1.4mg/dl | P29, blood cfDNA, EMseq, single library | 99697 | CGGAAT | 1,321,615,851 | **399.1** | 2,643,231,702 | 2,142,823,336 | 103.7 | 91.34 | 35.47 |
|  |  | P29, urine cfDNA, EMseq, single library | 99696 | CACCGG | 1,173,833,146 | **354.5** | 2,347,666,292 | 1,766,694,050 | 85.5 | 89.87 | 35.15 |
|  | n/a; donor | P29, donor WGS | 98392 | CGTTAGAA+TTCAGGTC | 213,755,717 | **64.6** | 427,511,434 | 366,581,728 | 17.7 | 92.12 | 35.62 |

Table S8: Sequencing and associated clinical data for each sample in this study, replication cohort.

| **Sample name** | **Clinical data** | **Sample description** | **Sample ID** | **Barcode sequence** | **PF Clusters** | **Yield (Gbases)** | **PE reads for alignment** | **Uniquely mapped reads** | **Estimated hg19 coverage** | **% >= Q30**  **bases** | **Mean Quality**  **Score** |
| --- | --- | --- | --- | --- | --- | --- | --- | --- | --- | --- | --- |
| K19 | Healthy  Adult male | K19, blood cfDNA, EMseq | 115150 | TGATAGGC+GTCTGAGT | 364,781,559 | **110.2** | 729,563,118 | 596,098,448 | 28.8 | 90.97 | 35.41 |
|  |  | K19, blood cfDNA, Bisulfite conversion | 115133 | CACTGTAG+AAGCGACT | 868,501,224 | **262.3** | 729,563,118* | 565,730,072 | 27.4 | 91.58 | 35.52 |
| K20 | Healthy  Adult male | K20, blood cfDNA, EMseq | 115151 | ACGAATCC+ATTACCCA | 482,087,641 | **145.6** | 964,175,282 | 782,437,800 | 37.9 | 91.40 | 35.47 |
|  |  | K20, blood cfDNA, Bisulfite conversion | 115134 | GTGCACGA+TGATAGGC | 772,226,816 | **233.2** | 964,175,282* | 748,270,032 | 36.2 | 91.39 | 35.48 |
| K21 | Healthy  Adult male | K21, blood cfDNA, EMseq | 115152 | GTCTGAGT+GACTTGTG | 430,295,348 | **129.9** | 860,590,696 | 710,488,340 | 34.4 | 92.17 | 35.63 |
|  |  | K21, blood cfDNA, Bisulfite conversion | 115135 | AAGCGACT+ACGAATCC | 776,245,622 | **234.4** | 860,590,696* | 668,588,714 | 32.4 | 91.10 | 35.43 |
| P30 | End-stage renal disease on chronic peritoneal dialysis due to previous episode of atypical haemolytic uremic syndrome (aHUS)  Extrarenal disease:  Acute necrotizing pancreatitis,  elevated liver enzymes  Adult female | P30, blood cfDNA, EMseq | 115136 | ATTACCCA+CACTGTAG | 372,761,942 | **112.6** | 745,523,884 | 613,852,730 | 29.7 | 91.74 | 35.53 |
| P31 | Polycystic kidney disease of unknown aetiology,  slowly progressive chronic kidney disease  Adolescent female | P31, blood cfDNA, EMseq | 115139 | GACTTGTG+GTGCACGA | 348,541,758 | **105.3** | 697,083,516 | 562,076,074 | 27.2 | 90.78 | 35.36 |
| P32 | End-stage renal disease on chronic haemodialysis due to Primary Hyperoxaluria type I  Extrarenal disease:  Bone oxalosis  Adult female | P32, blood cfDNA, EMseq | 115140 | TTCAATAG+TCCCACGA | 390,065,125 | **117.8** | 780,130,250 | 642,975,044 | 31.1 | 91.62 | 35.51 |

*: the larger bisulfite-converted datasets were downsampled to the size of the corresponding EMseq dataset prior to alignment.

**Supplementary References**

1. Moss J, Magenheim J, Neiman D, Zemmour H, Loyfer N, Korach A, Samet Y, Maoz M, Druid H, Arner P, et al: **Comprehensive human cell-type methylation atlas reveals origins of circulating cell-free DNA in health and disease.** *Nat Commun* 2018, **9:**5068.

2. Lo YM, Chan KC, Sun H, Chen EZ, Jiang P, Lun FM, Zheng YW, Leung TY, Lau TK, Cantor CR, Chiu RW: **Maternal plasma DNA sequencing reveals the genome-wide genetic and mutational profile of the fetus.** *Sci Transl Med* 2010, **2:**61ra91.

3. Wang JC: **Helical repeat of DNA in solution.** *Proc Natl Acad Sci U S A* 1979, **76:**200-203.

4. Cheng THT, Jiang P, Tam JCW, Sun X, Lee WS, Yu SCY, Teoh JYC, Chiu PKF, Ng CF, Chow KM, et al: **Genomewide bisulfite sequencing reveals the origin and time-dependent fragmentation of urinary cfDNA.** *Clin Biochem* 2017, **50:**496-501.
